# Developmental emergence of sparse and structured synaptic connectivity in the hippocampal CA3 memory circuit

**DOI:** 10.1038/s41467-026-71914-x

**Published:** 2026-04-21

**Authors:** Victor Vargas-Barroso, Jake F. Watson, Andrea Navas-Olive, Alois Schlögl, Peter Jonas

**Affiliations:** Institute of Science and Technology Austria (ISTA), Klosterneuburg, Austria

**Keywords:** Synaptic transmission, Development of the nervous system, Neural circuits, Computational neuroscience

## Abstract

Hippocampal CA3 pyramidal neurons (PNs) form the largest autoassociative network in the mammalian brain. Whether CA3–CA3 recurrent connectivity is genetically preconfigured or environmentally shaped during ongoing memory storage is currently unknown. To address this question, we performed multicellular patch-clamp-based circuit mapping of up to eight CA3 PNs in the mouse hippocampus at multiple postnatal time points (P7–8, P18–25, and P45–50). Here, we show that the hippocampal CA3 network undergoes a developmental transformation from local, dense, and random connectivity to a distributed, sparse, and structured configuration. Thus, sparse and structured connectivity may emerge via experience-dependent mechanisms. In parallel, the strength of single synapses is downregulated; single synaptic events are sufficient to trigger postsynaptic spiking early in development, whereas spatial summation of several inputs is required at later time points. Biologically inspired models of memory storage by Hebbian synaptic plasticity and retrieval via pattern completion suggest that developmental changes improve specific aspects of memory storage and retrieval. Our results imply a developmental transformation of the neuronal code and the memory functions in the hippocampal CA3 network.

## Abstract (web summary)

Using multicellular patch-clamp recording, the authors show that the hippocampal CA3 autoassociative network undergoes pruning of CA3–CA3 synapses and downregulation of synaptic efficacy during postnatal development, suggesting a double transformation of coding properties and memory functions.

## Introduction

The hippocampus plays a key role in the encoding, storage, and recall of spatial and nonspatial information^1–3^. In the center of the hippocampal trisynaptic circuit, CA3 pyramidal neurons (PNs) connect to one another, forming the largest autoassociative network throughout the brain^4–7^. This autoassociative network is thought to play a critical role in the storage and recall of information^8–10^. During memory storage, the activation of a subset of CA3 PNs increases synaptic weights of CA3–CA3 recurrent collateral synapses by Hebbian plasticity^11,12^ or behavioral time-scale plasticity^13^. During memory recall, potentiated synapses mediate the retrieval of original patterns from incomplete or degraded versions through the process of pattern completion^6,14–17^. Thus, the hippocampal CA3 circuit is thought to enable storage of information through plastic recurrent synapses, and at the same time support information recall through pattern completion, implementing a powerful content-addressable memory system.

Several lines of evidence suggest that the hippocampal CA3 network is a sparse but broadly connected network^4,6,7,18^. How the connectivity of this circuit emerges during postnatal development remains unclear. In a *tabula rasa* model of synaptic connectivity^19^, functional connectivity would be initially sparse and gradually increase as information is stored in the network. In contrast, in a pruning model, connection probability would start out at a high level and then decline, resulting in sparser but more specific innervation patterns^20,21^. Thus, *tabula rasa* and pruning models make different predictions of how connectivity will change over developmental time.

Recent work also suggests that synaptic connectivity in mature hippocampal and neocortical networks is not random, but enriched in disynaptic and higher-order connectivity motifs^6,22–24^. How these features are generated is unclear. One possibility is that network motifs are genetically preconfigured. For example, clonally related cells^25^ or neurons born at the same developmental time^26–28^ might be preferentially connected. In this scenario, nonrandom features of connectivity might be more pronounced at early developmental time points. Alternatively, changes in connectivity might be driven by functional and structural plasticity mechanisms^11–13^. In this framework, connectivity motifs may represent previously stored information (engrams), and therefore would be expected to emerge with experience over time. Again, genetic and plasticity mechanisms predict different trajectories of connectivity during postnatal development.

CA3–CA3 synapses play a key role in pattern completion and memory retrieval^6,14,15,29^. However, CA3–CA3 synapses in the mature circuit are weak, with excitatory postsynaptic potential (EPSP) amplitudes in the submillivolt to millivolt range^6,18,30^. Thus, several EPSPs have to be integrated by temporal and spatial summation to generate action potentials (APs) in a postsynaptic target cell^31^. How temporal and spatial summation emerge during postnatal development is unknown. On the one hand, synaptic strength may increase through spike timing-dependent plasticity^11,12,32^, or behavioral time-scale synaptic plasticity^13^, increasing the impact of individual synapses. On the other hand, synaptic strength may decrease by homeostatic mechanisms^33^. Furthermore, the effective strength of a synapse is not only influenced by synaptic conductance but also by dendritic location, passive cable properties, and excitability of the postsynaptic cell. Consistent with this idea, excitability has been shown to bias engram allocation in several brain circuits^34–36^. Whether the excitability of CA3 cells changes during postnatal development is unclear, and how many active inputs are needed to generate postsynaptic spiking at different developmental time points is unknown.

In this work, we measured functional connectivity in the mouse CA3 network using octuple multicellular patch-clamp recordings in early postnatal (P7–8), juvenile (P18–25), and mature (P45–50) animals. These postnatal stages are particularly interesting because they correspond to time points before, during, and after the critical period of plasticity in sensory systems^37^, and cover the sensitive period for episodic memory formation^38^. We found decreasing connection probability in the CA3 recurrent network during postnatal development, and an overabundance of non-random disynaptic connectivity motifs only in the mature circuit. These findings support a pruning model of network maturation, in which experience continuously shapes the circuit. In parallel, we observed a surprising switch in the properties of synaptic integration, with detonating transmission occurring in CA3 early in development and a requirement for spatial summation and coincidence detection emerging at later stages, consistent with a sparse coding mechanism. In combination with modeling, our results suggest that modifications at the connectomic, cellular, and synaptic levels shift the CA3 recurrent memory system into an optimal functional state. Preliminary accounts of our work were previously published in abstract form (Vargas-Barroso, Watson and Jonas, 2022, Society Neurosci Abst 034.11/C39.2022).

## Results

### Sparsification of synaptic connectivity in the developing mouse CA3 network

**Fig. 1:**
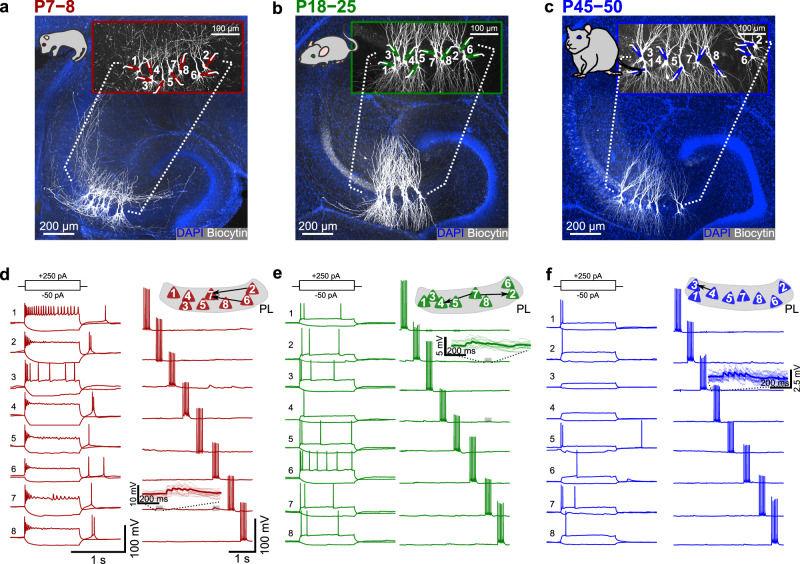
Multicellular patch clamp-based analysis of functional connectivity in the mouse CA3 network at three developmental time points. **a–c** Representative low magnification maximum intensity projections of simultaneously recorded PNs filled with biocytin and visualized using Alexa Fluor 647-conjugated streptavidin, across developmental stages (P7–8, P18–25, and P45–50). Inset, higher magnification maximum intensity projections depicting the detailed arrangement of recorded cells and corresponding recording pipettes (schematics). In total, 70, 77, and 81 multicellular recordings were performed. **d–f** Representative traces of octuple patch-clamp recordings for each age. The AP phenotype (left) of each PN in response to current injections (−50 and +250 pA shown only) is characterized. Average traces (20–30 sweeps, right) in CC configuration of the sequential, simultaneous stimulation protocol for each cell, used to screen for functional connections. Identified connections are highlighted (gray boxes) and shown at expanded time/amplitude scales in insets (only one connection for each recording shown for simplicity). Schematic of recording configurations and connections found, shown above; PL: pyramidal layer of CA3. Scale bars in the main panels are the same for the three age groups. Source data are provided as a Source Data file.

**Fig. 2:**
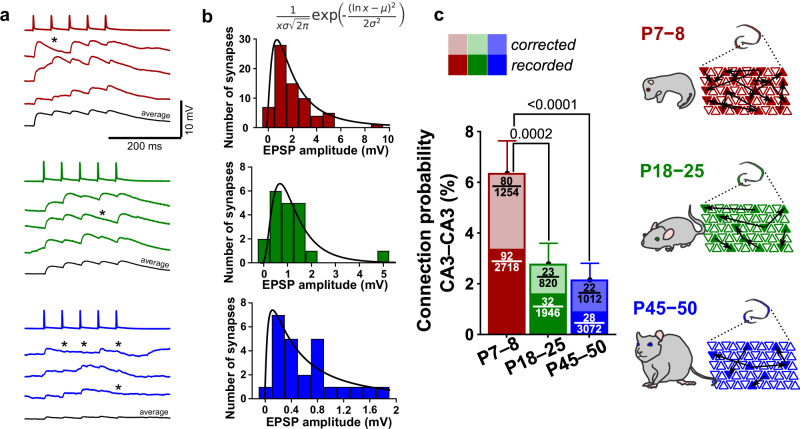
Developmental decrease in the connection probability among CA3 PNs. **a** Example connections recorded from the three age groups. Presynaptic APs elicited by current injection with corresponding postsynaptic responses (EPSPs) of monosynaptically connected neurons, average response shown in black. Asterisks depict transmission failures (peak amplitude of the response less than three times the standard deviation of the preceding baseline). **b** Frequency distributions for the peak amplitude values of the first EPSP of P7–8, P18–25, and P45–50 synapses. Curves represent log-normal distribution functions (equation shown on top) fit to the measured distributions. EPSP peak amplitudes were significantly higher in P7–8 synapses than in later developmental time-points; *p* = 0.0155 for P7–8 vs. P18–25; *p* = 0.2908 for P18–25 vs. P45–50; *p*  <0.0001 for P7–8 vs. P45–50. Reported *p* values were obtained from non-parametric Kruskal–Wallis test, followed by two-sided Dunn’s multiple comparisons test. **c** Left, connection probability within the CA3–CA3 network across postnatal development. Bar graphs show recorded (dark colors) and corrected (light colors) connection probabilities for each age group. Numbers inside the bars show the number of found (numerator) and tested (denominator) connections for each condition and age group. Error bars indicate standard deviations obtained from a binomial distribution. Error bars calculated from a binomial distribution. Two-sided Fisher’s exact test, *p* = 0.002 for P7–8 vs. P18–25; *p* = 0.02 for P18–25 vs. P45–50; *p* < 0.0001 for P7–8 vs. P45–50, *p* values of pairwise comparisons after Benjamini-Hochberg correction. Right, schematic representation of the developmental decline of connection probability among CA3 PNs. Source data are provided as a Source Data file.

To probe possible changes in synaptic connectivity in the CA3 network across postnatal development, we performed simultaneous, multi-cellular patch-clamp recordings from CA3 PNs in acute hippocampal slices from P7–8, P18–25, and P45–50 mice (Figs. 1 and 2). To keep synaptic connectivity intact as much as possible, we aimed to record from PNs with somata ≥50 µm below the surface of the slice. PNs were targeted based on their location within the CA3 pyramidal layer and morphological appearance in the infrared-differential interference contrast (DIC) video image. For rigorous identification, cells were filled with biocytin during recording and visualized using 3,3’-diaminobenzidine (DAB) or fluorescently-conjugated streptavidin (see Methods for details). In total, we tested 7736 connections (P7–8: 2718; P18–25: 1946, and P45–50: 3072; see Supplementary Fig. 1 for additional information). To probe functional connectivity, we stimulated putative presynaptic PNs in the current-clamp configuration by eliciting five APs at a frequency of 20 Hz and recorded both EPSPs (current clamp, CC) and excitatory postsynaptic currents (EPSCs, voltage clamp, VC; Supplementary Fig. 2) in postsynaptically connected neurons (Fig. 1d–f). Frequency distribution histograms of monosynaptically connected CA3 PNs showed a skewed distribution of EPSP amplitude, disclosing a high degree of variability of synapse strength across development (Fig. 2a, b).

We first determined a measured CA3–CA3 connection probability as the number of found over tested synaptic connections (Fig. 2c). Altogether, the measured connection probability was 3.38 ± 1.12% for P7–8, 1.64 ± 0.63% for P18–25, and 0.91 ± 0.58% for P45–50. These results suggest that the CA3–CA3 synaptic interconnectivity declines with age. We next computed a corrected connection probability, including only PNs as presynaptic partners in which the axon was largely preserved (Supplementary Fig. 3; inclusion criterion for axons: ≥500 µm of total axon)^7^. Altogether, the corrected CA3–CA3 connection probability was 6.37 ± 1.28% at P7–8, 2.8 ± 0.81% at P18–25, and 2.17 ± 0.65% at P45–50 (Fig. 2c; *p* = 0.002 for P7–8 vs. P18–25 and *p* < 0.0001 for P7–8 vs. P45–50; Fisher’s exact test). These results corroborate our conclusion that synaptic interconnectivity in hippocampal CA3 declines during postnatal development. We also analyzed connectivity values within age groups and found no statistical differences, revealing homogenous properties within our chosen developmental time windows (Supplementary Fig. 4).

To further characterize the properties of synaptic connectivity across development, we analyzed the dependence of connection probability on intersomatic distance (Supplementary Fig. 5a−f). In early postnatal CA3, distance-dependent connection probability was apparent within distances of 0 to 375 µm (Supplementary Fig. 5d, g). In contrast, consistent with previous observations in the mature rat^6^, in P18–25 and P45–50 age groups, connection probability was more constant in the distance range examined (Supplementary Fig. 5e–g). Thus, the CA3 network shifts from a more local to a more distributed connectivity during postnatal development. To further examine whether the developmental reduction in connectivity differed along the proximodistal axis, we compared connectivity between CA3a/b (the CA3 subfield close to the CA2 region) and CA3c (the CA3 subfield close to the dentate gyrus (DG)) at P7–8, P18–25, and P45–50. Connection probability was not significantly different between CA3a/b and CA3c at any of these time points, suggesting that the developmental reduction in connectivity equally affected all three CA3 subfields (Supplementary Fig. 5h, i). Taken together, our results show a substantial developmental decrease in the connection probability between PNs during maturation, consistent with a pruning model of CA3 network development.

### Morphological factors underlying sparsification of synaptic connectivity

**Fig. 3:**
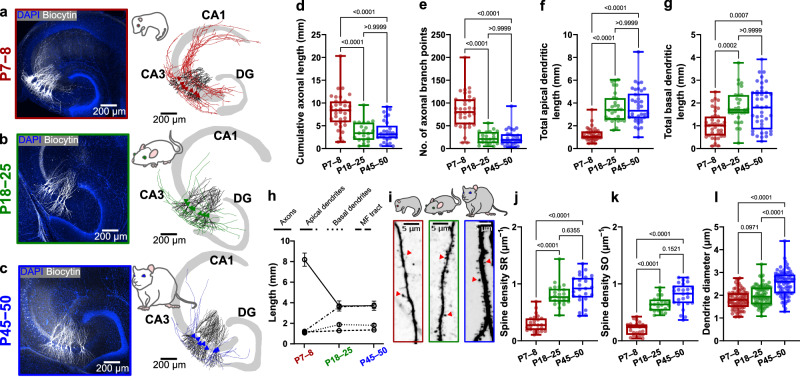
Axonal pruning predicts synaptic connectivity. **a–c** Representative maximum intensity projections of biocytin-streptavidin visualized PNs (left) and 3-D reconstructions (right) of CA3 PNs from the three developmental time points studied. Only PNs with preserved axons are depicted. Axons and somata are shown in red, green, and blue for P7–8, P18–25, and P45–50, respectively; dendrites are shown in black. Hippocampal subfields (DG, CA3, and CA1) were delineated based on DAPI staining. Micrograph in (**c**) same as in Fig. 1c. **d, e** Box plots showing morphological analysis of reconstructed axons. Axonal parameters show a significant reduction of total length per cell (**d**) and number of branch points (**e**), suggesting axonal pruning. Cumulative axonal length: mean ± SEM; P7–8: 8.13 ± 0.64 mm, *n* = 35 cells; P18–25: 3.69 ± 0.49 mm, *n* = 21 cells; P45–50: 3.7 ± 0.45 mm, *n* = 29 cells. Number of axonal branch points: mean ± SEM; P7–8: 83.6 ± 6.39, *n* = 35 cells; P18–25: 24.14 ± 2.98, *n* = 21 cells; P45–50: 24.00 ± 3.42, *n* = 29 cells. **f, g** Box plots showing morphological analysis of reconstructed dendrites. Dendrites in P7–8 pups were not fully developed. Total apical (**f**) dendritic length: mean ± SEM; P7–8: 1.12 ± 0.9 mm, *n* = 39 cells; P18–25: 3.60 ± 0.25 mm, *n* = 28 cells; P45–50: 3.70 ± 0.25 mm, *n* = 38 cells. Total basal (**g**) dendritic length: mean ± SEM; P7–8: 1.06 ± 0.9 mm, *n* = 39 cells; P18–25: 1.8 ± 0.13 mm, *n* = 28 cells; P45–50: 1.80 ± 0.15 mm, *n* = 38 cells. **h** Plots showing absolute change (mean ± SEM) of length for mossy fiber tract, CA3 axons, apical dendrites, and basal dendrites, throughout postnatal development; P7–8, *n* = 39 cells; P18–25, *n* = 33 cells; P45–40, *n* = 45 cells. **i** Representative high-resolution micrographs of dendritic spines (red arrowheads) for each age group. **j–l** Box plots showing quantification of dendritic spine density of apical (SR) and basal (SO) dendrites on PNs in all age groups, as well as corresponding dendritic diameter. Spine density of apical dendrites: mean ± SEM; P7–8: 0.30 ± 0.02 μm^−1^, *n* = 24 cells; P18–25: 0.83 ± 0.03 μm^−1^, *n* = 24 cells; P45–50: 0.90 ± 0.05 μm^−1^, *n* = 25 cells. Spine density of basal dendrites: mean ± SEM; P7–8: 0.20 ± 0.02 μm^−1^, *n* = 23 cells; P18–25: 0.64 ± 0.02 μm^−1^, *n* = 23 cells; P45–50: 0.80 ± 0.04 μm^−1^, *n* = 25 cells. Dendritic diameter: mean ± SEM; P7–8: 1.80 ± 0.04 μm, *n* = 76 dendrites; P18–25: 1.98 ± 0.04 μm, *n* = 77 dendrites; P45–50: 2.51 ± 0.06 μm, *n* = 77 dendrites. Reported *p* values were obtained from non-parametric Kruskal–Wallis test, followed by two-sided Dunn’s multiple comparisons test. Box plots show: points, individual data; whiskers, minimum to maximum range; box, 25–75% range; horizontal line, median. Source data are provided as a Source Data file.

Changes in synaptic connectivity could be caused by alterations in axonal or dendritic density^39^. To distinguish between these possibilities, we analyzed the axonal arbors and dendritic trees of CA3 PNs (Fig. 3). A subset of biocytin-filled CA3 PNs was labeled using fluorescently conjugated streptavidin, imaged using a spinning-disk confocal microscope, and morphologically reconstructed (Fig. 3a–c). Consistent with the idea of developmental pruning, but contrary to what might be expected from a growth process, we found that the cumulative axonal length within the transverse slice plane became significantly reduced as the network developed (Fig. 3d). Accordingly, we observed a decrease in the number of axonal branch points (i.e., collateral ramifications; Fig. 3e). Notably, axonal remodeling and length reduction were mostly confined to CA3, given that pruning was mainly observed within a distance of ~400 µm from PNs somata, as revealed by Sholl analysis (Supplementary Fig. 5m). Therefore, pruning of the local axonal arborization appears to be a specific feature of CA3 microcircuit refinement during postnatal development.

To test whether changes in dendritic configuration contributed to connectivity changes, we performed a similar analysis of the dendritic arborization. In contrast to the axonal arbor, dendritic length significantly increased during postnatal development, from P7–8 to older age groups. These changes were similar for both apical and basal dendrites (Fig. 3f, g). Additionally, we compared axonal and dendritic length with the length of the mossy fiber tract as a proxy of hippocampal growth. While the growth-trajectory of CA3 PN dendrites closely followed that of the mossy fiber tract, only axons showed a paradoxical decrease in length (Fig. 3h). To further investigate the mechanisms for the observed decline in functional synaptic connectivity, we quantified spine density in CA3 PNs during postnatal development (see Methods). We found a striking developmental increase in spine density and, in parallel, a slight increase in dendritic diameters (Fig. 3i–l). Thus, not only the dendritic length, but also the spine density increased during postnatal development. In conclusion, the developmental changes in connectivity are highly asymmetric. Whereas CA3 axons undergo a substantial pruning process during postnatal development, CA3 dendrites show continuous growth, reflected by an increase in dendritic length and spine density.

### Morpho-functional transition from random to structured network organization

**Fig. 4:**
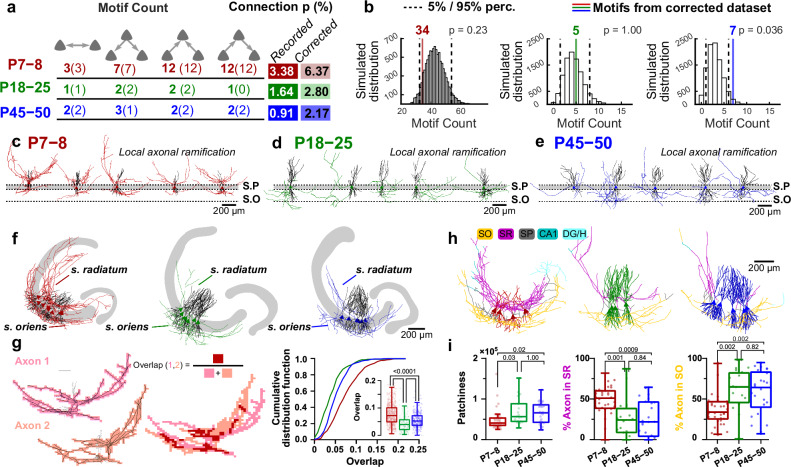
The CA3 recurrent network undergoes a morpho-functional shift from a random to a structured configuration. **a** Motif count and connection probability matrix of recorded and corrected data sets. Left, number of reciprocal, divergence, convergence, and chain synaptic motifs found in each age group. Values obtained in the recorded (dark colors) and corrected data sets (light colors) are shown. Right, connection probabilities of recorded and corrected data sets. **b** Connected cell identities were randomly simulated for all recorded data sets (10,000 simulations), and random motif abundance was quantified (distributions). The black vertical lines indicate 5th and 95th percentiles, while red, green, and blue vertical lines indicate the number of experimentally recorded motifs for P7–8, P18–25, and P45–50, respectively. The number of motifs is significantly larger than that expected by chance only in the mature network (right). Motif analysis was performed using corrected connection probability and the number of motifs. **c–e** P7–8 PNs exhibit stereotypical axonal morphology. Single cell reconstructions from five different animals (**c**), depicting highly similar axonal morphologies (i.e., stereotypical ramification patterns). Single-cell reconstructions from five different animals (P18–25 and P45–50; **d, e**), depicting more variable axonal morphology (no stereotypical ramification pattern). Neuronal processes are color-coded as in Fig. 3. **f** Multicellular reconstructions from P7–8 recordings show homogenous axonal distribution, blanket-covering both *stratum radiatum* and *stratum oriens*; in contrast, multicellular reconstructions from P18–25 and P45–50 recordings show more patchy, non-uniform axonal distributions. **g** Left, schematic representation of the axonal overlap (stereotypy) for two P7–8 PNs from different animals. Right, cumulative plot of the probability density function for axonal overlap across development. Inset, axons of P7–8 PNs show significantly higher degree of overlap (stereotypy) in axonal ramification patterns (box plots). P7–8, *n* = 39 cells; P18–25, *n* = 33 cells; P45–40, *n* = 45 cells. **h** Representative reconstructions of recordings in which the axons of all cells were segmented to quantify the percentage of axons targeting different layers/subfields (color-coded): *stratum pyramidale* (SP), *stratum oriens* (SO), *stratum radiatum* (SR), CA1, and dentate gyrus / hilus (DG/H). **i** Left: box plots showing significantly higher degree of axonal patchiness in PNs of older animals. P7–8, *n* = 39 cells; P18–25, *n* = 33 cells; P45–40, *n* = 45 cells. Axon patchiness was computed using Ripley’s K function; for analysis of overlap and patchiness, a more stringent criterion for inclusion of axons was used (see Methods). Center, right, box plots showing the percentage of axon targeting SR and SO for each age group. SR proportion decreases, whereas SO proportion increases with age (Supplementary Fig. 6). Reported *p* values obtained from a non-parametric Kruskal–Wallis test, followed by two-sided Dunn’s multiple comparisons test. Box plots show: points, individual data; whiskers, minimum to maximum range; box, 25–75% range; horizontal line, median. Source data are provided as a Source Data file.

We next sought to determine whether the observed synaptic and axonal modifications were related to changes in the network’s morpho-functional organization (Fig. 4). In several neocortical and hippocampal subcircuits, connectivity appears to be non-random, enriched in reciprocal, convergence, divergence, and chain motifs^6,22,23^. To test whether the enriched presence of these motifs is a genetically determined trait of the network or if it is acquired by experience, we compared the recorded number of disynaptic motifs to simulated data, randomizing the identity of connected cells in each dataset^40^ and utilizing average connection probabilities and number of motifs from our corrected connectivity data (Fig. 4a). While the number of recorded disynaptic motifs was within the possible range for random connectivity at P7–8 and P18–25, the mature network (P45–50 age group) showed a greater number of recorded motifs than expected by chance (*p* = 0.03; Fig. 4a, b). Thus, structured connectivity appears to emerge in CA3 over development, consistent with a possible experience-dependent process.

Next, we asked whether the development of structured connectivity was associated with structural changes in the axonal configuration. To address this question, we analyzed inter- and intra-group variability of the morphological properties of the axonal arborization (Fig. 4c−e). To quantify the variability of the axonal morphology among cells, we measured the overlap of the axonal distribution of the aligned cells of each group using the Intersection-over-Union method (see Methods; Fig. 4f, g). This analysis revealed that the degree of axonal overlap was significantly higher in the P7–8 age group than in P18–25 and the P45–50 age groups (*p* < 0.0001; Fig. 4f, g). Thus, local axonal arborizations were more stereotypical at early developmental time points but more variable from cell to cell at later stages.

To quantify the variability of axonal structure within a given cell, we computed the amount of axon patchiness using Ripley’s K function^41^. A stringent cell inclusion criterion was applied, only considering PNs with cumulative axon length longer than 1500 µm. This analysis revealed that early in development, axonal arborizations were more uniform, whereas later in development, axon ramifications acquired more patchy distributions (Fig. 4i). Thus, the patchiness of the axonal arborization of CA3 PNs, also reported earlier^4^, was not present at early developmental time points but was acquired later in postnatal development. Are axon collaterals at later time points pruned from specific target subregions/layers? To address this question, we performed segmentation analysis to quantify the axonal distribution in the following target areas: *stratum pyramidale* (SP), *stratum oriens* (SO), *stratum radiatum* (SR), CA1, and DG/Hilus (DG/H). We found that the distribution of axon collaterals within CA3 significantly changed during postnatal development. In the early postnatal period, the proportions of axon collaterals in SR and SO were similar, whereas in the juvenile and adult periods, the SO component became more prominent (Fig. 4h and Supplementary Fig. 6). Altogether, the functional transition of the CA3 autoassociative network from dense-random to broad-structured was associated with axonal morphological changes, such as loss of stereotypy and patchy appearance.

### Switch from near-detonation to synaptic integration

**Fig. 5:**
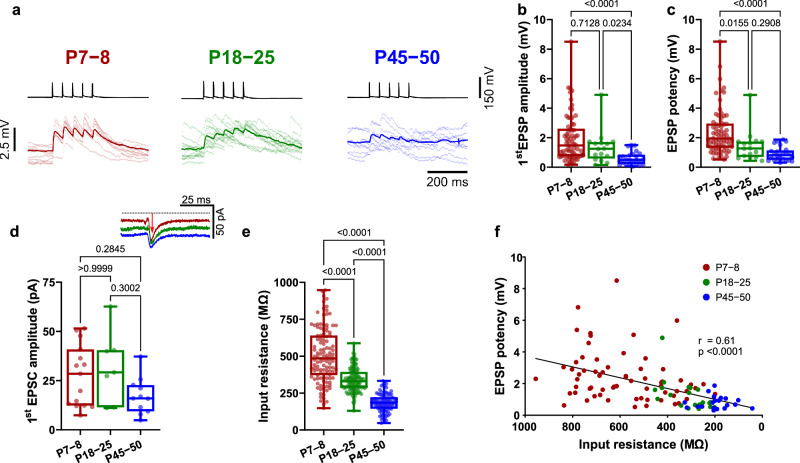
Unitary EPSP amplitude of CA3–CA3 synapses significantly decreases throughout development. **a** Representative monosynaptic EPSP responses to a train of five presynaptic APs (20 Hz) for each age group (dark and light traces are single and average responses, respectively). **b, c** Box plots of unitary EPSP amplitude and potency (amplitude of successes) show a statistically significant reduction of synapse strength throughout development. EPSP peak amplitude: mean ± SEM; P7–8: 1.94 ± 0.18 mV, *n* = 70; P18–25: 1.34 ± 0.27 mV, *n* = 16; P45–50: 0.59 ± 0.08 mV, *n* = 25. EPSP potency: mean ± SEM; P7–8: 2.39 ± 0.18 mV, *n* = 70; P18–25: 1.43 ± 0.23 mV, *n* = 16; P45–50: 0.90 ± 0.09 mV, *n* = 25. **d** No significant changes were observed in EPSC amplitudes across development (only pairs with *R*_s_ < 20 MΩ were considered; inset shows representative EPSC responses for each age group). EPSC amplitude: mean ± SEM; P7–8: 27.5 ± 4 pA, *n* = 15; P18–25: 31.7 ± 6.8 pA, *n* = 7; P45–50: 17.2 ± 2.8 pA, *n* = 11. **e** The input resistance of PNs significantly decreases throughout development. Input resistance: mean ± SEM; P7–8: 506 ± 17 MΩ, *n* = 116; P18–25: 338 ± 7 MΩ, *n* = 123; P45–50: 184 ± 7 MΩ, *n* = 91. In **b**–**e,** *p* values were obtained from non-parametric Kruskal-Wallis tests, followed by two-sided Dunn’s multiple comparisons test. Box plots show: points, individual data; whiskers, minimum to maximum range; box, 25–75% range; horizontal line, median. **f** EPSP potency is significantly correlated with input resistance. Spearman rank correlation analysis shows a significant correlation between the two variables: *r* = 0.61, *p* < 0.0001. Data were further analyzed by linear regression (black line). Source data are provided as a Source Data file.

The developmental changes towards sparser and more structured connectivity may imply that different synaptic properties are required to initiate activity (i.e., to allocate PNs to a given ensemble). To explore this possibility, we examined the properties of synaptic transmission across the three age groups (Fig. 5). To assess the functional impact of single synapses, we measured peak amplitudes of single unitary EPSPs in connected pairs of PNs. We found that unitary EPSP peak amplitude and potency (i.e., the amplitude of successes) were highest in the P7–8 age group, but significantly declined with age (Fig. 5a–c). These results suggest a double transformation of coding properties of the CA3 network: not only did the local connectivity become sparser, but also the amplitude of unitary EPSPs became smaller.

**Fig. 6:**
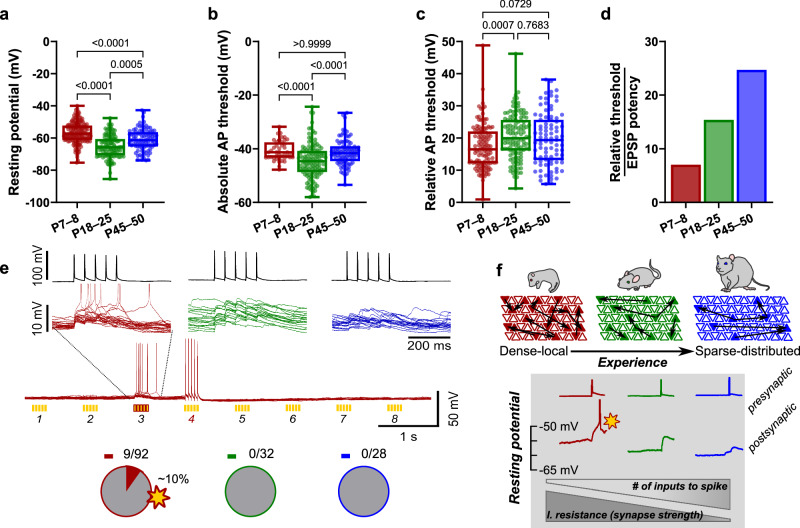
Detonating CA3–CA3 synapses in the P7–8 network. **a** Box plots of resting potential throughout development. Resting potential: mean ± SEM; P7–8: −56.72 ± 0.51 mV, *n* = 166; P18–25: −65.24 ± 0.62 mV, *n* = 123; P45–50: −61.08 ± 0.69 mV, *n* = 91. **b** Box plots of absolute AP threshold across development. AP threshold: mean ± SEM; P7–8: −40.76 ± 0.55 mV, *n* = 43; P18–25: −44.66 ± 0.58 mV, *n* = 123; P45–50: −41.29 ± 0.51 mV, *n* = 89. **c** Box plots of relative AP threshold (difference between absolute AP threshold and resting potential) throughout development. Relative threshold: mean ± SEM; P7–8: 17.37 ± 0.61 mV, *n* = 121; P18–25: 20.57 ± 0.62 mV, *n* = 123; P45–50: 19.72 ± 0.86 mV, *n* = 89. In **a**–**c,** *p* values were obtained from non-parametric Kruskal–Wallis tests, followed by two-sided Dunn’s multiple comparisons test. Box plots show: points, individual data; whiskers, minimum to maximum range; box, 25–75% range; horizontal line, median. **d** Bar plots depicting the ratio of relative threshold to average EPSP potency across development. **e** Top, detonating synapses were only observed in P7–8 synapses, whereas identical stimulation paradigms for P18–25 or P45–50 connections failed to elicit APs in postsynaptic cells. Center, example membrane voltage traces of a P7–8 PN (cell 4) showing a detonating response (first set of APs) to stimulation of a monosynaptically connected presynaptic neuron (cell 3). In addition, cell 4 was activated by direct current injection (second set of APs). Yellow vertical bars indicate stimulation times of all PNs in the recording; bars with red contours indicate the detonating synapse. Bottom, proportion of synapses (~10%) in P7–8 connections that showed detonating properties; no detonating synapses were recorded in P18–25 or P45–50 pairs. **f** Schematic of the morpho-functional re-arrangement of the CA3 PN network. Top, transition of the network from a highly locally connected network to a sparsely, broadly distributed network; these changes might be shaped by experience. Bottom, mechanisms that result in the changes of detonation properties of CA3–CA3 synapses during postnatal development. A significant decrease in input resistance of PNs across development (determinant of synapse strength), along with an increase in the number of inputs required to reach AP threshold, results in a shift from near-detonation to spatial summation (see Supplementary Fig. 8) in the CA3 autoassociative network. Source data are provided as a Source Data file.

To determine the underlying mechanisms, we analyzed the properties of corresponding EPSCs (Fig. 5d). Unitary EPSC peak amplitude was slightly, but not significantly smaller in the mature synapses. Thus, changes in EPSC properties do not predict the EPSP amplitudes. Given that the active and passive properties of CA3 PNs showed significant changes during postnatal development (Supplementary Fig. 7), we further tested the hypothesis that changes in input resistance may underlie the developmental reduction in EPSP amplitude^42^. Indeed, input resistance significantly decreased across age groups (Fig. 5e) and was significantly correlated with synaptic potency in the total data set (Fig. 5f; Spearman correlation, *r* = 0.61, *p* < 0.0001). Thus, a cell-specific mechanism regulates the peak amplitude of unitary EPSPs. To determine the number of active inputs necessary for AP generation, and potentially ensemble allocation, we compared the amplitudes of single unitary EPSPs with the relative threshold for AP initiation (see Methods; Fig. 6). The ratio of amplitude of single unitary EPSPs to relative AP threshold, an estimate of the number of unitary synaptic events required to reach AP threshold, increased from ~7 to ~15 and finally ~25 during postnatal development (Fig. 6a–d), indicating a growing requirement for dendritic integration to elicit APs.

AP threshold could be reached by either temporal or spatial summation^31^, and both forms of synaptic integration might be developmentally regulated. To test for temporal summation, we applied trains of five stimuli at 20 Hz in the presynaptic CA3 PNs (Fig. 6e). Although synapses in the P7–8 age group were significantly more depressing (Supplementary Fig. 8d, e), the EPSP decay was slower (Supplementary Fig. 8c) and temporal summation was more prominent (Fig. 6e). Unexpectedly, in ~10% of tested pairs in the immature network, stimulation of a single presynaptic neuron resulted in the initiation of APs in the postsynaptic neuron during train stimulation, consistent with previous observations in young guinea-pigs^30^. In contrast, in both the P18–25 and the P45–50 age groups, postsynaptic firing did not occur (P18–25: 0 out of 32 pairs; P45–50: 0 out of 28 pairs; five stimuli at 20 Hz; Fig. 6e). These results indicate that synaptic transmission shifts from near-detonation in the early CA3 network to temporal and spatial summation in the mature network. It is widely believed that detonation is a specific property of large synapses, such as hippocampal mossy fiber synapses^43^. Our results demonstrate that more conventionally sized synapses also show detonation features in the young hippocampal circuit.

Finally, we tested the properties of spatial summation in recording configurations in which two presynaptic neurons converged on the same postsynaptic target neuron (Supplementary Fig. 8a). In all age groups, responses to simultaneous activation of two convergent inputs were comparable to the arithmetic sum of the individual responses (4 convergence motifs in the P18–25 age group, 1 convergence motif at P18–25; 1 convergence motif at P45–50; Supplementary Fig. 8b). However, quantitative analysis of the summation ratio (EPSP_PN1+PN2_/(EPSP_PN1_ + EPSP_PN2_)) suggested a slight shift from sublinearity to supralinearity during postnatal development (*p* = 0.03; two-way ANOVA; Supplementary Fig. 8b). Together with the acceleration of membrane time constant (Supplementary Fig. 7c) and EPSP decay time constant (Supplementary Fig. 8c), these results suggest a developmental shift from temporal summation to spatial summation and coincidence detection in CA3 PNs^44,45^.

### Developmental transformation optimizes memory retrieval in a CA3 network model

**Fig. 7:**
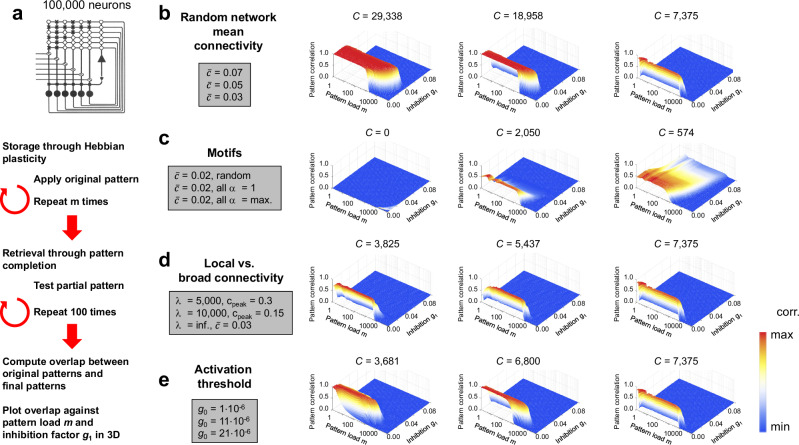
Developmental transformation of synaptic connectivity and efficiency optimizes memory capacity in a CA3 network model. **a** Schematic illustration of the network model (from ref. ^29^ with permission of Elsevier). Large circles, CA3 somata; triangle, inhibitory interneuron; small circles, potentiated CA3–CA3 synapses; small crosses, unpotentiated synapses; horizontal lines on left, mossy fiber inputs. **b** Effects of average synaptic connectivity in a random network. Left, c̅ = 0.07; center, c̅ = 0.05; right, c̅ = 0.03. 3D plots represent overlap between retrieved and original patterns (vertical axis), plotted versus pattern load (*m*, number of patterns stored in the network, front axis) and strength of global inhibition (*g*_1_, right axis). Color code according to scale bar (bottom right). **c** Effects of structured versus random connectivity on memory retrieval by pattern completion. Left, 3D plot for reduced average synaptic connectivity (c̅ = 0.02). Note failure of memory retrieval. Center, 3D plot for low average synaptic connectivity (c̅ = 0.02) in combination with nonrandom connectivity motifs (*α*_recip_ = *α*_conv_ = *α*_div_ = *α*_chain_ = 1). Inclusion of nonrandom connectivity motifs, as experimentally observed in the mature network, rescued memory retrieval. Right, 3D plot for low average synaptic connectivity (c̅ = 0.02) in combination with maximally enhanced abundance of nonrandom connectivity motifs. **d** Effects of local versus broad connectivity. Left, 3D plot for local synaptic connectivity, as observed in young networks (*λ* = 5000; peak connection probability *c*_peak_ = 0.3). Center, similar 3D plot for intermediate synaptic connectivity (*λ* = 10,000; peak connection probability *c*_peak_ = 0.15). Right, similar 3D plot for broad synaptic connectivity, as observed in mature networks (uniform connection probability c̅ = 0.03). Total mean connection probability was the same in all cases. Developmental shift from local to broad connectivity increased memory capacity. **e** Effects of strong-detonating versus weak-integrating synapses on memory retrieval by pattern completion. Strength of the synapses was controlled via the activation threshold parameter *g*_0_. Left, 3D plot for strong synapses, as observed in the young network. In these simulations, *g*_0_ = 1 × 10^−6^, corresponding to a scenario where a single synapse is sufficient to trigger postsynaptic spiking. Center, similar 3D plot for *g*_0_ = 11 × 10^−6^, i.e., two synapses required for spiking. Right, similar 3D plot for *g*_0_ = 21 × 10^−6^, i.e., three synapses required for spiking. Developmental shift from strong near-detonating to weak integrating synapses increases memory capacity. In all panels, numbers indicate estimated memory capacity values. Source data are provided as a Source Data file.

Our results from multicellular patch clamp-based circuit mapping indicate a transformation in both synaptic connectivity (Figs. 2 and 4) and unitary synaptic transmission (Figs. 5 and 6) during postnatal development. To test how these developmental changes affect higher-order computations in hippocampal CA3, we assembled a biologically inspired autoassociative memory network model^6,7,15,46^ (Fig. 7). The model was implemented in full-size, with 100,000 neurons, consistent with the number of CA3 neurons in one hemisphere of the mouse hippocampus^47^. Storage of patterns was accomplished via a clipped Hebbian synaptic plasticity rule^46^, and recall was simulated as iterative pattern completion (Fig. 7a; see Methods). Random binary patterns were used because CA3 receives decorrelated information from the DG.

We first analyzed the effects of changes in synaptic connectivity in a randomly connected network (Fig. 7b). As expected, a gradual decrease of total average connection probability c̅ from 7 to 5 and 3% decreased memory capacity, as the total number of synapses became reduced. In contrast, when the number of synapses per cell was kept constant, the dependence of memory capacity on c̅ became saturating (Supplementary Fig. 10b). Thus, sparse connectivity can increase the efficiency of memory storage and retrieval, albeit when implemented in a non-physiological way in which the total number of cells was altered.

Next, we tested the effects of nonrandom connectivity features (Fig. 7c). In a random network in which connectivity was further reduced to 2%, memory retrieval failed; hence, memory capacity was close to zero under these conditions (Fig. 7c, left). Introducing nonrandom connectivity features (α values for reciprocal, convergence, divergence, and chain motifs all set to 1) restored retrieval (Fig. 7c, center). Further increasing α values to the maximum possible values widened the area of successful retrieval in the pattern load (*m*)–inhibition (*g*_1_) parameter space, although memory capacity was reduced (Fig. 7c, right). These results corroborate the previous conclusion that nonrandom connectivity motifs enhance memory retrieval in sparsely connected networks^6,48^.

Our experimental results demonstrate a shift from local to broad connectivity (Supplementary Fig. 5). To address how such a change affects the function of the autoassociative memory network, we implemented distance-dependent connectivity in the model (Fig. 7d). Distance dependence was implemented via changes in the length constant (*λ*), while peak connectivity was inversely scaled with *λ* to keep the total connectivity constant at a value of 3%. Notably, network models with local connectivity gave lower memory capacity, while network models with broad connectivity showed higher capacity (Fig. 7d). Thus, the developmental transformation from dense, local connectivity to sparse, broad connectivity enhanced the memory performance of the network.

Our experimental data reveal a developmental transformation from strong near-detonating towards weak integrating synapses (Figs. 5 and 6). To test how these differences affect memory capacity, we globally changed synaptic efficacy by modifying the activation threshold *g*_0_. This parameter was changed between 1 × 10^−6^, 11 × 10^−6^, and 21 × 10^−6^, corresponding to one, two, and three synapses required to initiate activity in postsynaptic cells, respectively (Fig. 7e). Intriguingly, developmental transformation from strong-detonating synapses to weak-integrating synapses, with all other parameters kept constant, resulted in a substantial increase in memory capacity (Fig. 7e, right). Taken together, these results indicate that developmental transformation of connectivity and synaptic properties optimize the memory capacity of the CA3 network (Fig. 7), while leaving pattern separation largely intact (pairwise pattern analysis; see Methods; Supplementary Figs. 9 and 10).

## Discussion

The autoassociative CA3 network plays a key role in encoding, storage, and recall of spatial and nonspatial information^6,8–10,49^. Yet, how the properties of the CA3 network emerge during postnatal development is poorly understood. We have studied the CA3 circuit at three developmental time points, which cover critical events in the development of sensory systems, including the onset of hearing (P12) and eye opening (P14)^37^, as well as the sensitive period for episodic memory formation (P20–P24)^38^. Our results provide several insights into the structure, function, and developmental plasticity of this important brain network. First, we found that local connectivity decreased during postnatal development, suggesting a pruning mechanism (Fig. 2). Thus, the CA3 network starts from a *tabula plena* rather than a *tabula rasa* configuration. Functional pruning was accompanied by sparsification of axonal arbors, but growth of dendritic length and spine density, implying asymmetry in the pruning process (Fig. 3). Second, we observed an overabundance of non-random connectivity motifs only in the mature CA3 network. This functional reorganization was accompanied by profound local axonal remodeling, with a shift from uniform-stereotypic to more patchy-variable distributions (Fig. 4). Thus, the network transitions from a dense-random to a sparse-structured configuration. Finally, we found that the rules of synaptic integration underwent a substantial transformation during postnatal development. In the young CA3 circuit, subsets of synapses were unexpectedly strong, close to the threshold of AP initiation. Thus, temporal summation was sufficient to generate spikes. In contrast, in the mature circuit, ~25 unitary synaptic events were needed to reach the firing threshold under our experimental conditions (Figs. 5 and 6). Thus, spatial summation and coincidence detection become increasingly important. Taken together, our results reveal fundamental changes in connectivity, morphology, and synaptic integration in the CA3 network during postnatal development.

How synaptic connectivity in complex neuronal networks is generated during postnatal development remains enigmatic. It is often thought that connectivity in circuits is established by a pruning mechanism, as proposed in the neocortex^20,21,50^. However, there are also various examples where targeted growth mechanisms are involved in circuit development^51^. Our findings in hippocampal CA3 appear to be more consistent with a pruning mechanism. A decline in connection probability is found for both measured and corrected connectivity values. However, these values will still represent local connectivity in the transverse slice, and cannot detect long-range longitudinal or contralateral projections^4,52^. Furthermore, dorso-ventral connectivity gradients might exist^53^. In principle, pruning could occur at both axonal and dendritic sites^50^. However, our results reveal a developmental reduction of axonal density, but a continuous growth of dendritic arbors and spines. Thus, the pruning process is asymmetric, mainly affecting axonal arborizations^39^. Both an increase in the proportion of long-range connections and an increase in the number of spine over shaft synapses may contribute to this asymmetry^54^. Interestingly, pruning affects the SR more than SO (Supplementary Fig. 6). This may be important for separating different inputs to CA3 pyramidal cells during network maturation^43^.

The molecular and cellular mechanisms underlying the pruning of synapses are not very well understood. It is possible that pruning is a temporal extension of synaptic depression from the functional to the structural level^55^. Microglia are also likely to be involved^56^. Finally, parvalbumin-positive (PV^+^) GABAergic interneurons may play a role^57^. These neurons undergo significant maturation during postnatal development^58^ and control the consolidation of early memories^59^. Thus, they also may affect pruning and the emergence of structured connectivity.

Our functional analysis corroborates the view that synaptic connectivity in the CA3 network is not random^6^, and further shows that this feature is under developmental control (Fig. 4). Connectivity appears to be random in the young network, but becomes enriched in disynaptic connectivity motifs at later times. In parallel, our structural analysis demonstrates that the axonal distribution changes, being more uniform and stereotypic in the young group and more patchy and variable from cell to cell in the mature group. Thus, the whole network undergoes a morpho-functional transition from random to structured. Accumulating evidence suggests that synaptic connectivity in different circuits is non-random^6,22–24^. The mechanisms by which connectivity motifs emerge remain elusive. Recordings in the neocortex indicate that neurons generated from the same precursor cell are preferentially connected^25^. Furthermore, experiments in the hippocampus suggest that neurons born around the same time could be preferentially coupled^26^. Alternatively, connectivity motifs may arise as a consequence of synaptic plasticity. As the relative abundance of connectivity motifs increases during postnatal development (Fig. 4b), our results seem more consistent with the second mechanism. Thus, sparse and structured connectivity might be part of the engram, i.e., the cellular, molecular, and structural changes underlying learning and memory^60^. Motifs may interact with early connectivity and pruning in different ways: they may be generated from previously connected cells by selective elimination, or from unconnected cells by selective formation. Thus, sparse and structured connectivity may result from the combination of pre-programmed and experience-dependent mechanisms. Longitudinal imaging at the single-synapse level could be used to distinguish between these possibilities.

Development not only affects connectivity but also fundamentally changes the properties of synaptic integration (Figs. 5 and 6). Integration of synaptic potentials in dendrites can occur by two mechanisms: temporal and spatial summation^31^. Several mechanisms may promote temporal summation in young animals, including the slow EPSP decay time constant and abundant burst firing of CA3 neurons in vivo^61,62^. In contrast, in the mature circuit, spatial summation becomes increasingly important, resulting in coincidence detection properties of the circuit (Supplementary Fig. 8). The latter might be crucially relevant, given that in vivo, CA3 PNs fire in synchrony during network oscillations^63^ (M. Picher and P.J., unpublished). Thus, the network may switch from integration to coincidence detection. This could contribute to the developmental decline of the average firing rate of hippocampal neurons (Supplementary Fig. 7)^64–66^. Conversely, our observations may explain why the young hippocampus is more prone to seizures^67^. It is widely thought that the depolarizing action of GABA explains the seizure susceptibility of the young hippocampus^68^. Our results suggest that both high connectivity and detonation properties of young CA3–CA3 synapses also play important roles.

How do developmental changes in CA3 PNs and synapses affect higher-order computations in the hippocampus, such as spatial coding or pattern completion^17^? Changes in connectivity of the CA3 network may contribute to the developmental refinement of the spatial map, which involves an increase in the proportion of place cells and a rise in spatial coherence during maturation^64–66^. Changes in connectivity may also explain the ability of the hippocampus to generate temporal sequences of neuronal activity, which become more frequent during postnatal development^69^. Changes in excitability, including a decrease in input resistance, an increase in relative AP threshold, and a decrease in sag amplitude, may reduce network activity and increase the threshold for incorporation of neurons into engrams. Thus, older networks may store engrams with higher precision and specificity. This idea is supported by research in CA1 showing that large engrams lead to imprecise memory representations in juvenile mice (~P20) and that smaller engrams form more precise memory representations close to adulthood (~P60)^70^.

Our biologically inspired model provides further insights into how developmental changes contribute to the optimization of memory capacity and higher-order computations in autoassociative networks (Fig. 7). First, a development-like reduction in average connectivity can, under certain conditions, increase memory capacity. Second, a switch from more local to more distributed connectivity almost doubles memory capacity. Third, the change in the activation threshold from one to two or three synapses required for spiking increases memory capacity by almost a factor of two. Finally, changes in the abundance of nonrandom connectivity motifs enhance retrieval success rate in sparsely connected networks, confirming previous observations in mechanistic models based on biological observations^6^. Similarly, in normative models in which storage capacity is optimized, sparse connectivity and reciprocal or divergence motifs emerge as characteristic features^48^. Thus, development-like changes improve different aspects of memory storage and retrieval^6,71^. Taken together, these findings suggest that multiple postnatal developmental changes converge on the optimization of hippocampal CA3’s memory function. Interestingly, changes in network parameters also affect the pattern similarity index obtained by pairwise pattern correlation analysis. Whereas changes in the width of connectivity appear to favor pattern completion, other changes may enhance pattern separation (Supplementary Fig. 10). However, changes in the upstream DG circuit, the primary area where pattern separation takes place, also need to be considered. In addition, gradients along the proximodistal axis need to be taken into account. It has been demonstrated that CA3c is more prone to pattern separation, whereas CA3a and b are more tailored to pattern completion^72,73^.

Do the present results obtained in rodents have any implications for our understanding of learning and memory in the human brain^7^? It is intriguing that human episodic memory undergoes highly specific changes during postnatal development^74^. Whereas semantic memory develops early, episodic memory develops relatively late, approximately two years after birth in humans. As this time point is thought to correspond to the developmental time point of 3 weeks in mice^38,74^, such changes may be related to the developmental phenomena investigated in the present paper. Somewhat paradoxically, memories may form early in development, but cannot be retrieved at later times, a phenomenon referred to as infantile amnesia^70^. The underlying neurobiological mechanisms are unclear, but might be related to the pruning of synapses in the hippocampal circuit. Finally, the properties of encoding and processing of information are not constant, but exhibit a switch from generalization to specificity as the circuit matures, a feature termed infantile generalization^75^. These changes have been interpreted as a transition of hippocampal higher-order computations from pattern completion to pattern separation^76^. Such changes could be related to transitions from dense-random to sparse-structured CA3 connectivity. Direct testing of these hypotheses will require more work in the human hippocampus^7^.

## Methods

### Animals

All procedures were performed in strict accordance with institutional, national, and European guidelines for animal experimentation, approved by the Bundesministerium für Bildung, Wissenschaft und Forschung of Austria. Wild-type C57BL/6J mice (RRID:IMSR_JAX:000664) of three age groups (P7–8, P18–25, and P45–50), both male and female, were used for experiments. All animals were housed with *ad libitum* access to food and water, under constant temperature (21 °C) and humidity (50–60%). Animals were kept under a 12-h light-dark cycle and used for experiments in the light phase. Cages contained mother, littermates, and complex nesting material (cotton-like and cardboard) to provide a sufficient level of enrichment. In older animals (P45–50), brains were extracted after transcardial perfusion under the ethical license “Connectomics” 2020-0.648.587 to P.J.

### Slice preparation and octuple recording in mouse hippocampus

Animals were anesthetized using isoflurane and subsequently sacrificed by decapitation. Obtaining high-quality slices, optimized for octuple patch-clamp recordings, is increasingly challenging as animals become older. Therefore, mice corresponding to the adult age (P45–50) were transcardially perfused with ice-cold high-sucrose artificial cerebro-spinal fluid (aCSF), containing: 64 mM NaCl, 25 mM NaHCO_3_, 2.5 mM KCl, 1.25 mM NaH_2_PO_4_, 10 mM D-glucose, 120 mM sucrose, 7 mM MgCl_2_, and 0.5 mM CaCl_2_ (osmolarity ~334 mOsm), equilibrated with 95% O_2_ and 5% CO_2_ gas mixture (carbogen). Brains were rapidly extracted into ice-cold high-sucrose aCSF solution. Typically, only the right hemisphere was used to obtain slices. A single sagittal cut was performed with a scalpel blade to separate the hemispheres. Hemispheres were glued (UHU superglue) with magic cut angles of α close to 0 and β close to –5°^77^ onto the specimen disc of a VT1200 semi-automatic vibrating blade microtome (Leica Microsystems). 350-μm-thick slices were cut from the central region of the hippocampus. Samples were oriented such that the cutting blade advanced from apical to basal dendrites of CA3 PNs. Comparison of Nissl/DAPI labeling in our own slices with Allen Brain Atlas DAPI stainings in 8- to 12-week-old mice revealed that slices were taken at a ventral-to-dorsal distance of ~4000–4500 μm. For a total ventral-to-dorsal axis length of ~10,000 µm, this indicates a bias towards the ventral hippocampus.

After cutting, slices were placed in a recovery chamber at 35 °C with continuous carbogen bubbling for ~30 min. Following recovery, slices were transferred to a customized recording chamber, and perfused with continuously bubbled aCSF (125 mM NaCl, 25 mM NaHCO_3_, 2.5 mM KCl, 1.25 mM NaH_2_PO_4_, 25 mM D-glucose, 2 mM CaCl_2_, and 1 mM MgCl_2_; osmolarity ~316 mOsm) for the remainder of the experiment. Patch pipettes were pulled from thick-walled borosilicate glass tubing (2 mm outer diameter, 1 mm inner diameter; Hilgenberg 1807542), and filled with an intracellular solution containing: 135 mM potassium D-gluconate, 20 mM KCl, 0.1 mM EGTA, 2 mM MgCl _2_, 2 mM Na _2_ATP, 0.3 mM NaGTP, and 10 mM HEPES, adjusted to pH 7.28 with KOH; osmolarity ~300 mOsm, with 0.2% (w/v) biocytin (Invitrogen). The resistance of the patch-pipettes when filled with intracellular solution was 3.5–5.5 MΩ. Pipettes were positioned manually with eight mini 25 micromanipulators (Luigs and Neumann) under visual control provided by infrared differential interference contrast (IR-DIC) videomicroscopy. Slices were secured with a platinum grid to avoid mechanical movement. Targeted cell bodies were located ~50–65 µm from the surface of the slice. CA3 PNs were identified on the basis of morphological appearance in the video-image, their position within the CA3 pyramidal layer, and the AP phenotype upon sustained current injection (family of 1-s long current injections from −100 pA to 400 pA, with 50 pA increments), with average firing frequencies of <20 Hz during the pulses. Along the radial axis, we focused on PNs with somata located in the superficial and central sublayers; cells in deep sublayers were avoided to minimize the probability of recording from deep athorny cells^78,79^. Along the transverse axis, neurons were recorded in all CA3 subfields (Supplementary Fig. 5h, i), with a slight preference for CA3b, a subregion with extensive recurrent collaterals^4,5^. The recording temperature was ~22 °C (range: 20–22 °C, room temperature).

Electrical signals were recorded using four Multiclamp 700B amplifiers (Molecular Devices). Signals were low-pass filtered with the built-in Bessel filter at 10 kHz and digitized at 20 kHz with a CED 1401 power3 AD/DA converter (Cambridge Electronic Design) connected to a personal computer. Pulse generation and data acquisition were performed using Signal software 6.0 (Cambridge Electronic Design) and custom-made stimulation-acquisition protocols. The presynaptic neuron was held in the CC mode and stimulated with a repetition interval of 5–10 s, unless differently specified. APs were elicited by brief current pulses (duration 1–2 ms, amplitude 1–2 nA) while the postsynaptic cells were held in either current- or voltage-clamp (CC or VC) configuration. For functional connectivity testing, we first performed a simultaneous CC recording for all neurons, sequentially stimulating each PN with ~300 ms time lags in a 4-s long sweep, 20–30 times (Fig. 1), allowing us to swiftly identify synaptically connected cells. Following initial testing under CC conditions, all neurons were subsequently stimulated individually in the CC mode, while the remaining cells were held in VC. This strategy allowed us to always focus first on the connected pairs (determined in CC) for the VC physiological characterization of CA3–CA3 postsynaptic responses. The presynaptic neuron under CC conditions was stimulated with a train of 5 current pulses (duration 1–2 ms, amplitude 1–2 nA, frequency 20 Hz), while keeping all the other neurons in the VC configuration at –70 mV. In total, 20–30 single traces were collected. A pair was judged to be monosynaptically connected if EPSCs and / or EPSPs were generated with a latency of <4 ms and/or a synaptic jitter of <0.4 ms at ~22 °C (see Supplementary Fig. 2f and Supplementary Fig. 5j–l), and had peak amplitudes of >2.2–2.5 times the standard deviation of the preceding baseline. Granule cell–granule cell (GC–GC) putative disynaptic connections were used to compare jitter values from polysynaptic connections to those of monosynaptic connections reported in this study (Supplementary Fig. 5l). In total, 7736 pairs of neurons were tested for chemical connectivity. For CC recordings, pipette capacitance and series resistance were compensated, and compensation was readjusted during the experiment when necessary. For VC recordings, series resistance was not compensated, but carefully monitored using 2-mV hyperpolarizing pulses every 6 s. For analysis of EPSC kinetics, series resistance was ≤20 MΩ (Supplementary Table 2). Membrane potentials reported in the text were not corrected for liquid junction potentials.

Total recording statistics were as follows. For P7–8: 70 recordings; 25 octuples, 18 septuples, 12 sextuples, 6 quintuples, 5 quadruples, and 4 triples, yielding a total of 2718 possible connections tested between CA3 PNs. For P18–25: 77 recordings; 13 octuples, 16 septuples, 8 sextuples, 3 quintuples, 19 quadruples, 10 triples, and 8 doubles, yielding a total of 1946 possible connections tested. Finally, for P45–50: 81 recordings; 24 octuples, 18 septuples, 23 sextuples, 10 quintuples, 5 quadruples, and 1 triple, yielding a total of 3072 possible connections tested.

### Labeling of pre- and postsynaptic neurons

Pre- and postsynaptic neurons were filled with biocytin (0.2%) during the recording period. After filling of pre- and postsynaptic neurons, pipettes were carefully withdrawn from their somata, typically resulting in the formation of outside-out patches at the pipette tips. Slices were either stained using 3,3’-diaminobenzidine (DAB) as chromogen or fluorescently labeled with Alexa Fluor-conjugated streptavidin (see below).

For DAB staining, slices were fixed for 12–24 h at 4 °C in a 0.1 M phosphate buffer (PB; 0.4 M NaH_2_PO + 0.4 M Na_2_HPO_4_, adjusted to pH 7.35; diluted finally to 0.1 M) solution containing 2.5% paraformaldehyde (PFA, TAAB Laboratories Equipment), 1.25% glutaraldehyde (GA, Carl Roth, 4157.1) and 15% (v/v) saturated picric acid solution (Sigma-Aldrich, P6744-1GA). After fixation, slices were treated with hydrogen peroxide (1%, 10 min, Sigma-Aldrich, 95321-100 ml) to block endogenous peroxidases, and rinsed in PB several times. Membranes were permeabilized with 1% Triton X-100 (Sigma-Aldrich) in PB for 1 h. Slices were then transferred to a phosphate-buffered solution containing 1% avidin-biotinylated horseradish peroxidase complex (ABC, Vectastain ABC-Elite Standard kit, Vector Laboratories PK-6100) and 1% Triton X-100 for ~12 h. Excess ABC was removed by several rinses in PB, and slices were developed in 0.036% DAB (Sigma-Aldrich, D5637-5G) with a 0.006% NiCl_2_ (Sigma-Aldrich, 223387-25 G)/0.008% CoCl_2_ (Sigma-Aldrich, C8661-25G) mixture for intensification and final addition of 0.01% hydrogen peroxide. Finally, slices were mounted on glass slides in Mowiol (Mowiol 4-88, Carl Roth, 713.2; dissolved in glycerol, Sigma-Aldrich, G-9012 and Tris-(hydroxymethyl)aminomethane, Sigma-Aldrich 252859 with HCl).

For fluorescent labeling, slices were fixed for 12–24 h at 4 °C in a 0.1 M PB solution containing 4% paraformaldehyde (PFA). After fixation, slices were washed with 0.1 M PB 3×, blocked and permeabilized 3× for 30 min in a PB solution containing 5% normal goat serum (NGS; Biozol, ENG9010-10) and 0.4% Triton in 0.1 M PB for 2–3 h. We then stained overnight with streptavidin conjugated to Alexa Fluor 647 (1:300; diluted from 2 mg ml^−1^ stock solution, Invitrogen S32357) in a PB solution containing 5% NGS and 0.4% Triton. The following day, slices were washed with PB 4× for 30 min and incubated in a 1 mg ml^−1^ stock solution of DAPI (4’,6-diamidino-2-phenylindole, dilactate) diluted 1:10,000 in PB (Invitrogen) for 10 min. Subsequently, slices were cleared by immersing them in CUBIC solution (50% sucrose, 25% urea, 10% 2,2'2”-nitrilotriethanol and 0.1% Triton X-100 in MilliQ water; all Sigma-Aldrich: 16104, U5128-500G, 90279-100 ml) at room temperature for ~10 min^80^. Finally, slices were rinsed once in fresh CUBIC solution and mounted in CUBIC surrounded by a ring of Mowiol.

### Imaging and 3-D reconstructions

Slices stained with DAB were imaged using an Olympus BX61 widefield microscope equipped with 4×, 10×, 20×, and 40× (water immersion) objectives. Pictures were taken using the in-built cellSense Dimension 1.6 software (Olympus, 2011). For fluorescently labeled slices, image acquisition was performed using an Andor Dragonfly confocal (dual spinning disk with 25 and 40 µm pinholes) microscope (Oxford Instruments) equipped with a Zyla 4.2 Megapixel sCMOS camera (2048 × 2048 pixels). Images were obtained either with a 10× air objective (Nikon MRD00105, CFI P-Apo 10×, NA 0.45) or a 20× water-immersion objective (Nikon P-Apo 20×, NA 0.95, WD 0.95 mm). Images were stitched with Imaris Stitcher software (Oxford Instruments), transformed to TIFF format, and transferred to the Neutube software for semi-automated reconstruction^81^. The resultant SWC files were used for morphological analysis (total axonal length, number of branches, and total apical and basal dendritic length) of reconstructed axons and dendrites using the SNT plug-in for Fiji^82^. Additionally, the axon of every reconstructed CA3 PN was segmented based on its distribution within the following hippocampal subregions or subfields: SR, SO, and SP within CA3, and CA1, DG, or hilus. We then obtained the proportion of every axon that traversed the aforementioned areas (Fig. 4 and Supplementary Fig. 6). For CA1, DG, and hilus, no subfield divisions were made. To account for mechanical compression of the slices due to the mounting procedure, we corrected the volume of the obtained reconstructions by scaling them to a thickness of 350 μm. SVG files of reconstructed neurons (single and multi-cell) were exported for further editing in Inkscape (Inkscape Project, https://inkscape.org). To unequivocally classify PNs, we used our electrophysiological profiling, as well as the unambiguous location and morphology of imaged neurons. We identified 290 (P7–8), 243 (P18–25), and 310 (P45–50) PNs, corresponding to 63%, 87%, and 59% of the recorded neurons for each age group, respectively.

### Spine density and dendritic thickness analysis

For spine counting, we obtained high-resolution images across postnatal development. For this purpose, we imaged fluorescently labeled slices using the Andor Dragonfly confocal microscope with a water-immersion 40× objective (Nikon MRD77410, Apochromat LWD lS 40×, NA 1.15) and manually segmented spines in five recordings for each age group, using Neutube. Apical and basal dendrites (dendrites with thorny excrescences excluded) corresponding to SR and SO inputs, respectively, were analyzed. The average dendritic length analyzed for apical dendrites was 433 ± 31 μm, for P7–8 (*n* = 24 cells), 430 ± 15 μm for P18–25 (*n* = 24 cells), and 427 ± 14 μm for P45–50 (*n* = 25 cells). The average dendritic length analyzed for basal dendrites was 333 ± 20 μm, for P7–8 (*n* = 23 cells), 351 ± 19 μm for P18–25 (*n* = 23 cells), and 362 ± 15 μm for P45–50 (*n* = 25 cells). The same images were used for measuring dendritic diameters across development. We analyzed the thickest portion of a given apical, primary dendrite along its Z-axis and measured 76, 77, and 77 dendritic diameters in P7–8, P18–25, and P45–50, respectively. Analysis was performed in 5 different animals/recordings (Supplementary Table 4). Comparison of spinning-disk confocal imaging data (this paper) and superresolution-expansion microscopy data in mouse CA3 PNs^7^ suggest that spine density may be underestimated by a factor of ~2, presumably due to the presence of spines emerging perpendicular to the slice plane, as well as to the resolution limit of the confocal microscope. However, our approach would still allow us to assess relative differences between age groups.

### Morphological analysis

All imaged neurons were analyzed in Fiji to obtain corrected connection probability values. Every identified PN of each recording was sorted and considered only as a potential postsynaptic partner (<500 μm of axon measured) or as both pre- and postsynaptic partner (≥500 μm of axon measured). Inter-somatic distances were measured as the Euclidean distance between somatic centers. Axonal Sholl analysis was performed using the SNT plug-in for Fiji^82^. Mossy fiber tract length was measured in Fiji by taking advantage of sporadic biocytin uptake from presynaptic mossy fiber terminals, which enabled visualization of the tract in a subset of slices of each age group studied (P7–8, *n* = 39 slices; P18–25, *n* = 33 slices; and P45–50, *n* = 44 slices). These measurements were used as a proxy for hippocampal growth across development (Fig. 3) and for approximate subregion parcellation of CA3 (Supplementary Fig. 5).

To analyze the axon overlap between recorded cells, we performed the following analysis. First, we aligned morphologies by centering the reconstructed coordinates in the soma. Cells were rotated so that the apical axis was parallel to the vertical axis. To find the apical axis direction, we input all the (x, y) apical coordinates to the PCA Matlab function and defined the slope as the direction of the first principal component. The rotation of the whole morphology was performed by multiplying all coordinates through the rotation matrix:

1 $${{\boldsymbol{R}}}=\left(\begin{array}{cc}\cos \varTheta & -\sin \varTheta \\ \sin \varTheta & \cos \varTheta \end{array}\right)$$

where *θ* = arctan (slope). The final alignment was such that the soma was located at (0,0) and the apical dendrite on the vertical axis below the soma. Second, we quantified the overlap of the axonal distribution of the aligned cells of each group using the Intersection-over-Union method. To achieve the latter, axons must be made spatially comparable, so we standardized the axonal morphology by transforming it into a 2D grid of 0 s and 1 s, depending on the presence (1) or absence (0) of an axon in each grid point. The grid was 140 × 140, with the *x* axis spanning from −700 μm to +700 μm every 10 μm, and the *y* axis spanning from −1000 μm to +400 μm every 10 μm. Figure 4g (left) depicts the transformation from the original axon coordinates (black lines) to the grid-like version (pink or salmon bins). For legibility purposes, illustrations used 20 μm, not 10 μm. Intersection over Union was computed using this grid representation of the axon. In particular, it was computed as the overlap area between the grids of any two neurons of each group (red area), divided by the total area covered by the two grids (pink, salmon, and red areas in Fig. 4g, right). For this analysis, only axons with total length >1500 μm were used (*n* = 34 for P7–8, *n* = 18 for P18–25, and *n* = 24 for P45–50).

To compute patchiness, we used Ripley’s *K* function^41^. Ripley’s *K* was computed as:

2 $$K=\frac{1}{n\lambda }{\sum }_{i}^{n}{\sum }_{j\ne i}^{n}({d}_{i,j} < t),$$

where λ is the average density of points and *t* is the radius around which to perform the analysis. Distances were computed over the standardized morphology (i.e., the above-described grid representation). To resolve a greater level of detail, we used a smaller bin size of 2 μm. The Ripley’s radius constant was set to *t* = 30 μm, but we observed similar significant tendencies for *t* ranging from 5 to 40 μm.

### Analysis of unitary EPSPs, EPSCs, and membrane properties

Unitary EPSPs and EPSCs were analyzed using Stimfit or equivalent Python-based scripts^83^. The rise time was measured as the time interval between the points corresponding to 20 and 80% of the peak amplitude. The peak of the EPSP or EPSC was determined as the maximum within a window of 1 or 2 ms duration, respectively, following the presynaptic AP. EPSP and EPSC potencies were calculated as the average amplitude of the successful responses without failures. In monosynaptically connected pairs, a trace was classified as a failure if the peak amplitude was less than three times the standard deviation of the preceding baseline. Synaptic latency was determined as the time interval between the peak of the presynaptic AP and the onset of the subsequent EPSP or EPSC; the onset point was determined from the intersection of a line through the 20 and 80% points with the baseline. The decay phases of the EPSPs or EPSCs were fit with a monoexponential function using a nonlinear least-squares fit algorithm.

The amplitude of the AP was measured as the peak voltage value from threshold. AP half-width was measured as the duration of the AP at half-maximal amplitude from threshold. In both cases, the first AP elicited by current injections was used for analysis. Apparent membrane time constant was measured by fitting an exponential function to the repolarization phase of the membrane potential immediately after hyperpolarizing current pulses. Input resistance was calculated using Ohm’s law from the steady-state portion of hyperpolarizing current injections to PNs. AP threshold was determined using depolarizing current steps; threshold was defined as the voltage at the point when the slope first exceeded a value of 20 V s^−1^. In a subset of recordings, threshold measurements were corroborated by ramp injections to cells followed by phase plot analysis, using the same dV/dt criterion. We counted the proportion of recorded PNs (all recorded neurons with regular firing patterns included) with rebound spikes, when present within 300 ms following the current injection step. d*V*/d*t*_max_ was calculated by fitting the first derivative of the somatic AP waveform. Sag potential was measured as a >1 mV (average) voltage difference between the most negative membrane voltage (first 200 ms of the 1-s long current injection) and the end of the pulse, during a hyperpolarizing stimulus of either −50 or −100 pA.

To determine the number of unitary synaptic events and inputs necessary to reach the firing threshold of a postsynaptic neuron, we computed the ratio of EPSP amplitude to relative threshold. Relative threshold was defined as the difference between the resting potential and the AP threshold (i.e., voltage displacement required to reach AP threshold). Resting potentials were measured immediately after the whole-cell configuration was obtained. To quantify the multiple-pulse ratio, >20 traces (including failures) were averaged. The amplitudes of the second and all subsequent EPSPs or EPSCs in the train were measured in average traces, setting the baseline directly before the onset of each synaptic event.

### Analysis of connectivity motifs

To test whether reciprocal, convergence, divergence, and disynaptic chain motifs^6,84^ occurred significantly more frequently than expected by chance, we simulated the entire set of recording configurations for each age group (P7–8, P18–25, and P45–50) 10,000 times, assuming random synaptic connectivity and average connection probability^85^. The number of recorded connections in each dataset was randomly assigned across all recordings, taking into account the identity of neurons considered as possible presynaptic partners (see corrected connectivity). Statistical *p* values were computed from CDF(*x*), where CDF is the cumulative distribution of motif number in the simulations, and *x* represents experimentally recorded numbers.

### Modeling of pattern completion in a CA3 network model

Simulations of pattern completion in autoassociative memory network models were performed closely following previous work^6,7,46^ (Supplementary Table 5). In the standard model, the total number of excitatory neurons *n* was 100,000, representing the CA3 pyramidal cell network of the mouse in one hemisphere^47^. To define the connectivity matrix *W* of size *n* × *n*, neurons were interconnected according to three different connectivity rules. First, neurons were connected randomly, assuming a uniform connection probability (c̅). Second, in a subset of simulations, reciprocal, convergence, divergence, and disynaptic chain motifs were included. To achieve this, we used a SONET algorithm^84^, setting *α*_recip_, *α*_conv_, *α*_div_, and *α*_chain_ to values >0, where α describes the enrichment of a given motif above the random level. Finally, in another subset of simulations, exponentially distance-dependent connectivity was implemented, with length constants of 5000 or 10,000 cells. To keep the total mean connectivity constant, peak connectivity was inversely scaled with the length constant, using a circular, linear neighborhood arrangement of neurons.

To simulate the storage of information, random activity patterns were applied to the network. Patterns were vectors of binary numbers of length *n*, in which 0 represented inactivity and 1 AP firing. The total average activity level *f* was assumed as 0.002, consistent with sparse activity of CA3 PNs in vivo^86^. For testing pattern separation and completion (Supplementary Fig. 10c), both random and correlated patterns were applied; correlated patterns were computed from multinormally distributed random numbers through thresholding. An increasing number (*m*) of random binary patterns was then loaded into the network. Synaptic plasticity was implemented using a clipped Hebbian rule^46^. Under this rule, when both pre- and postsynaptic neurons were simultaneously active during a pattern, synaptic weights were updated to 1; otherwise, weights were unchanged. The use of this rule was motivated by experimental data, demonstrating associative synaptic plasticity with symmetric induction rules at recurrent CA3–CA3 synapses^11–13^. Clipping of the synaptic weight at 1 ensured stability of the network.

To simulate iterative recall, 100 of the activity patterns originally used in the storage phase were reapplied in the retrieval phase. Patterns used for recall were degraded in comparison to the original ones applied during storage. The proportion of validly firing neurons (*b*_valid_) was 0.5, and the proportion of spuriously firing neurons (*b*_spurious_) was 0.001. We then simulated the ability of the network to iteratively recall the original patterns. Neuronal activity was simulated over 10 recall cycles. For each recall cycle, the total input to the ith neuron at time *t* was computed as

3 $${h}_{i}\left(t\right)=\frac{1}{n}{\sum }_{j=1}^{n}({{{\boldsymbol{W}}}}_{{ij}}{\cdot {{\boldsymbol{J}}}}_{{ij}}){{{\boldsymbol{X}}}}_{j}\left(t\right),$$

where ***W*** denotes the connectivity matrix, ***J*** represents the synaptic weight matrix, ***X***(*t*) is the network activity vector at time *t*, and “·” indicates element-wise multiplication.

During iterative recall, a neuron was assumed to fire APs at time *t* + 1 if the condition

4 $${h}_{i}\left(t\right)-{g}_{1}S\left(t\right) > {g}_{0}$$

was met, where $S(t)={\sum }_{j=1}^{n}{S}_{j}\left(t\right)$ is the total activity, *g*_*1*_ is the strength of global inhibition, and *g*_0_ is the firing threshold. *g*_*1*_ was varied between 0 and 0.1. In the default model, *g*_*0*_ was assumed as 21 × 10^−6^, corresponding to a scenario in which three synaptic inputs are required for AP initiation. More generally, *g*_*0*_ was set to (*N*_synapses_ – 1)/*n* + ε where *N*_synapses_ is the number of active synapses required to trigger spiking in a given neuron, *n* is the number of neurons (100,000), and ε = 1 × 10^−6^.

To quantify the accuracy of recall of a given pattern, pattern correlation (*r*) between original and recalled patterns was computed as the correlation coefficient between the corresponding vectors for the 10th recall cycle. Pattern correlation *r* was then plotted against the pattern load *m* and the strength of inhibition *g*_1_. These 3-dimensional *r*–*m*–*g*_1_ plots provided visual information about capacity, robustness of recall, and maximal correlation. Memory capacity *C* (i.e., number of patterns that could successfully be stored and retrieved) was quantified as the maximum of the product function of pattern correlation (*r*) and pattern load (*m*). To probe the pattern computations in the network during recall (i.e., pattern completion and pattern separation), we performed pairwise analysis of correlations between input and output patterns (Supplementary Fig. 9)^87^. Input patterns were defined as patterns used for storage, triggered by mossy fiber detonator synapses, whereas output patterns were taken as final patterns after the 10th recall cycle, dependent on CA3–CA3 synaptic activity. One-hundred patterns were analyzed, leading to *n*_pairs_ = $$\left(\begin{array}{c}100\\ 2\end{array}\right)$$ = 4950 pairwise comparisons. Pattern similarity was then assessed from the location of data points in the output–input correlation (*r*_out_–*r*_in_) plots in comparison to the identity line: points above the identity line indicate pattern completion, whereas points below the identity line suggest pattern separation^87,88^. To quantify the distance from the identity line, a pattern similarity metric was computed for each point as the perpendicular distance to the identity line, divided by the maximal possible distance, which was obtained by extrapolation of the perpendicular distance line towards the nearest axis. A pattern similarity index (ψ) was determined by averaging across all pairwise comparisons as:

5 $$\Psi \left({{\rm{x}}},{{\rm{y}}}\right)=\left({\sum }_{i=1}^{{n}_{{pairs}}}\left(y-x\right)/\min \left(\left|x\right|+\left|y\right|,2-\left|x\right|-{|y|}\right)\right)/{{{\rm{n}}}}_{{{\rm{pairs}}}},$$

where *x* and *y* represent *r*_in_ and *r*_out_, respectively, and | .. | denotes absolute values. If *x* values are zero, ψ will be +1 or −1, depending on whether elements are above or below the identity line. ψ values were then calculated for all grid points in the *m*–*g*_*1*_ surface, and a weighted average ψ was computed, using the corresponding overlap values in the overlap–*m*–*g*_*1*_ 3D plots (Fig. 7b–e) as weight factors.

Simulations of connectivity matrices, storage, and iterative recall were implemented in C or C++ and run on x86_64-based shared memory nodes, typically in dual socket configuration with at least 4 GB per CPU core, and CPUs from Intel or AMD with AVX2 or higher support. For computations, we used GNU/Linux (Debian12/Bookworm), the GNU C compiler (gcc/g++ 12.2), and the GNU scientific library (GSL 2.8), as well as the SLURM scheduling system and the LMOD module system for software management. Connectivity matrices containing nonrandom connectivity motifs were generated using the SONET simulation program package after minor improvements^6,84^. Modifications included optimization in memory management, storage of matrices in sparse format, and adaptation to a 64-bit computation platform. Final analysis was performed using Matlab (Mathworks; MATLAB R2024a) and Mathematica (Wolfram Research; 14.3). Code is available from 10.15479/AT-ISTA-21442 under the link https://research-explorer.ista.ac.at/download/21442/21443/ca3simu-vargas2026v1.tar.gz.

### Statistics and conventions

Values are given as mean ± standard error of the mean (SEM) in the text, legends, and tables. No statistical methods were used to predetermine sample size. Box plots show: points, individual data; whiskers, minimum to maximum range; box, 25–75% range; horizontal line, median. Unless specified differently, two-sided non-parametric statistical tests were used throughout the study. Statistical significance for three-way comparison of continuous data across age groups was examined using the non-parametric Kruskal-Wallis test, followed by Dunn’s multiple-comparison tests unless otherwise stated. Connection probabilities are represented as bars (measured data), with the standard deviations obtained from a binomial distribution. Differences in connection probabilities between age groups were tested for statistical significance using Fisher’s exact test, followed by individual pairwise comparisons with a Benjamini-Hochberg correction for multiple comparisons. Reported *p* values of pairwise comparisons were obtained after correction. Statistical tests and plotting were performed using GraphPad Prism 10; axonal distribution analysis and motifs analysis were done utilizing custom-made Matlab scripts; further analysis was performed using Matlab (Mathworks; MATLAB R2024a) or Mathematica (Wolfram Research; 14.3).

### Reporting summary

Further information on research design is available in the Nature Portfolio Reporting Summary linked to this article.

## Supplementary information

Supplementary Information

Reporting Summary

Transparent Peer Review File

## Source data

Source Data

## Data Availability

Source data are provided with this paper. Additional original data are available from the corresponding author upon request.

## Code availability

Code is available from 10.15479/AT-ISTA-21442 under the link https://research-explorer.ista.ac.at/download/21442/21443/ca3simu-vargas2026v1.tar.gz.
