# Supplementary material for: Developmental emergence of sparse and structured synaptic connectivity in the hippocampal CA3 memory circuit
Source: Nat Commun. 2026 Apr 21;17:5540. doi: 10.1038/s41467-026-71914-x (PMC13287488; doi:10.1038/s41467-026-71914-x)
Supplement: Supplementary file 1 — Supplementary Information [file 41467_2026_71914_MOESM1_ESM.pdf]

Developmental emergence of sparse and structured synaptic connectivity in the hippocampal CA3 memory circuit

Victor Vargas-Barroso[1], Jake F. Watson[1], Andrea Navas-Olive[1], Alois Schlögl[1], and Peter Jonas[1,*]

1 Institute of Science and Technology Austria (ISTA), Am Campus 1, A-3400 Klosterneuburg, Austria

* Corresponding author

Supplementary Figure 1 | Detailed information about recording configurations.

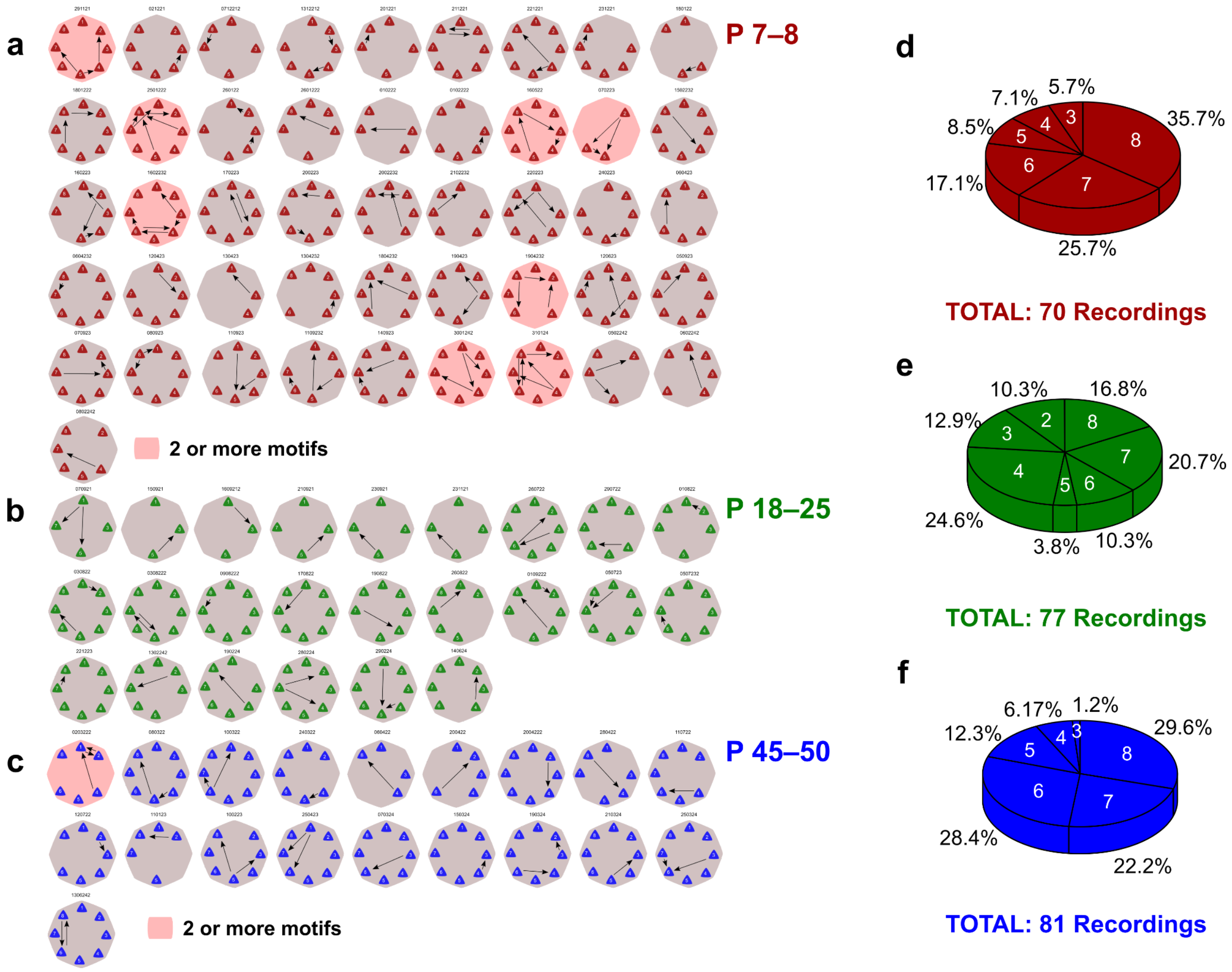

a–c Recording arrangements of every single recording in which synaptic connections were detected, for each age group studied. For all ages, the pink background represents recordings in which two or more disynaptic motifs were recorded. **d–f** Pie charts showing the proportion of octuples, septuples, sextuples, quintuples, quadruples, triples, and pairs of recorded neurons, for each age group, with the total number of recordings included in the dataset at the bottom.

Supplementary Figure 2 | Validation of monosynaptic connections in the voltage-clamp configuration and physiological properties of CA3–CA3 synapses throughout development.

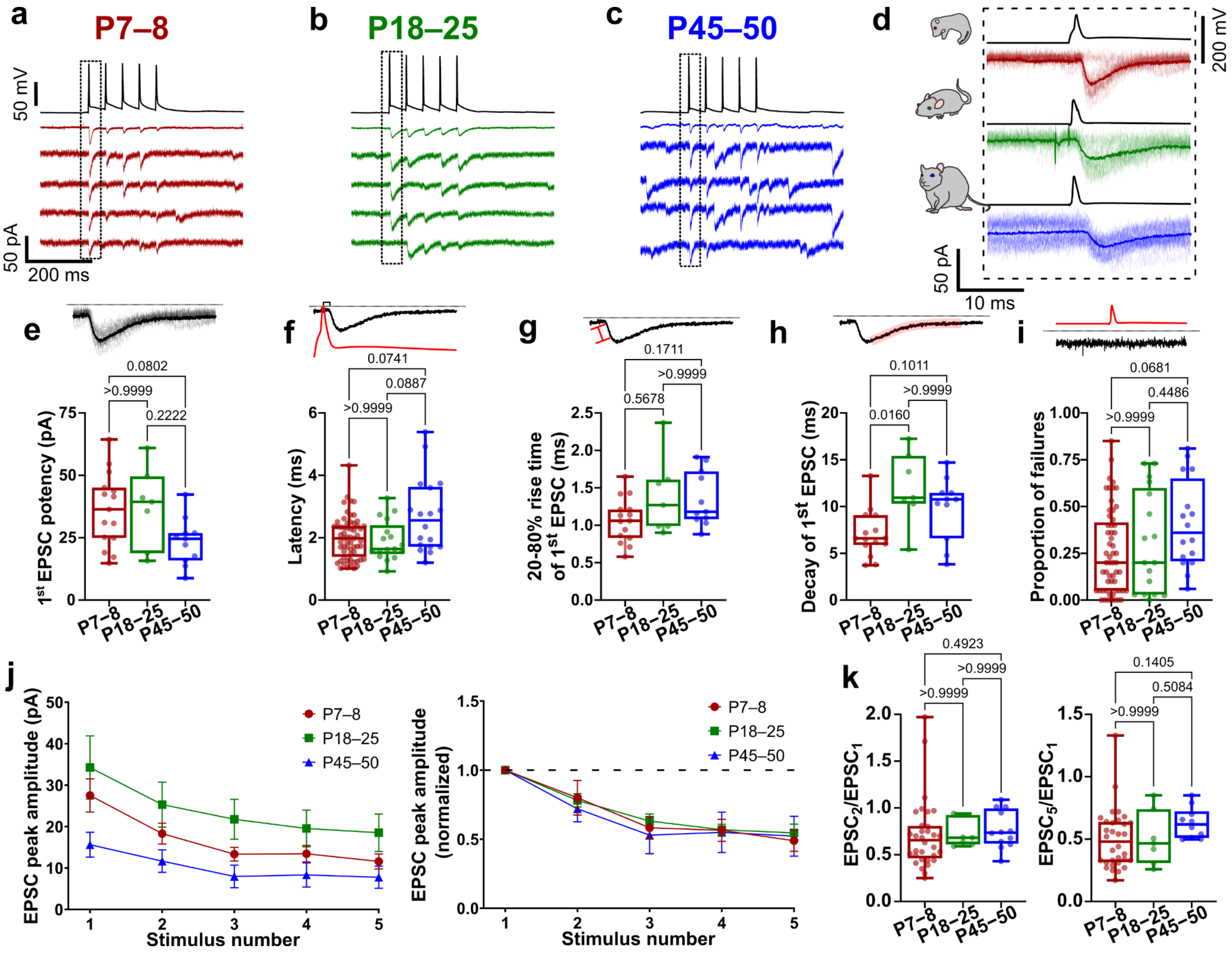

a–c Representative traces of monosynaptic connections recorded for the three age groups. All putative connections initially observed in the CC configuration were confirmed to be monosynaptic connections by subsequently recording EPSC responses in the voltage-clamp configuration. For each age group, presynaptic APs are shown at the top of panels (black) while average and individual traces, respectively, are shown below, color-coded accordingly. **d** Expanded time scales of representative unitary, monosynaptic EPSCs for each age (average and single traces). **e–i** Box plots (points, individual data; whiskers, min. to max.; box 25–75% values; horizontal line, median) of physiological properties of CA3–CA3 synapses. These properties remain largely constant throughout development. However, the EPSC decay time constant was significantly faster in synapses from P7–8 synapses, compared to P18–25 and P45–50. EPSC potency: mean ± SEM; P7–8: 36.16 ± 3.77 pA, *n* = 15; P18−25: 37.31 ± 6.04 pA, *n* = 7; P45−50: 23.36 ± 2.82 pA, *n* = 11. Latency: mean ± SEM; P7−8: 1.99 ± 0.08 ms, *n* = 60; P18−25: 1.90 ± 0.15 ms, *n* = 17; P45−50: 2.69 ± 0.28 ms, *n* = 18. 20–80% rise-time of first EPSC: mean ± SEM; P7−8: 1.06 ± 0.07 ms, *n* = 15; P18−25: 1.38 ± 0.19 ms, *n* = 7; P45−50: 1.35 ± 0.10 ms, *n* = 11. Decay time constant of first EPSC: mean ± SEM; P7−8: 7.04 ± 0.63 ms, *n* = 15; P18−25: 11.94 ± 1.47 ms, *n* = 7; P45−50: 9.81 ± 1.01 ms, *n* = 11. Proportion of failures: mean ± SEM; P7−8: 0.25 ± 0.02, *n* = 57; P18−25: 0.30 ± 0.06, *n* = 17; P45−50: 0.40 ± 0.05, *n* = 16. Reported *p* values obtained from non-parametric Kruskal-Wallis test,

followed by two-sided Dunn's multiple comparisons test. **j** Plots of EPSC peak amplitude and normalized EPSC amplitude ratio ($EPSC_n$ / $EPSC_1$) against stimulus number (n) for P7–8, P18–25, and P45–50 age groups, respectively. **k** Summary box plots of $EPSC_2$ / $EPSC_1$ and $EPSC_5$ / $EPSC_1$.

Supplementary Figure 3 | Correction of connection probability for severed axons in slices.

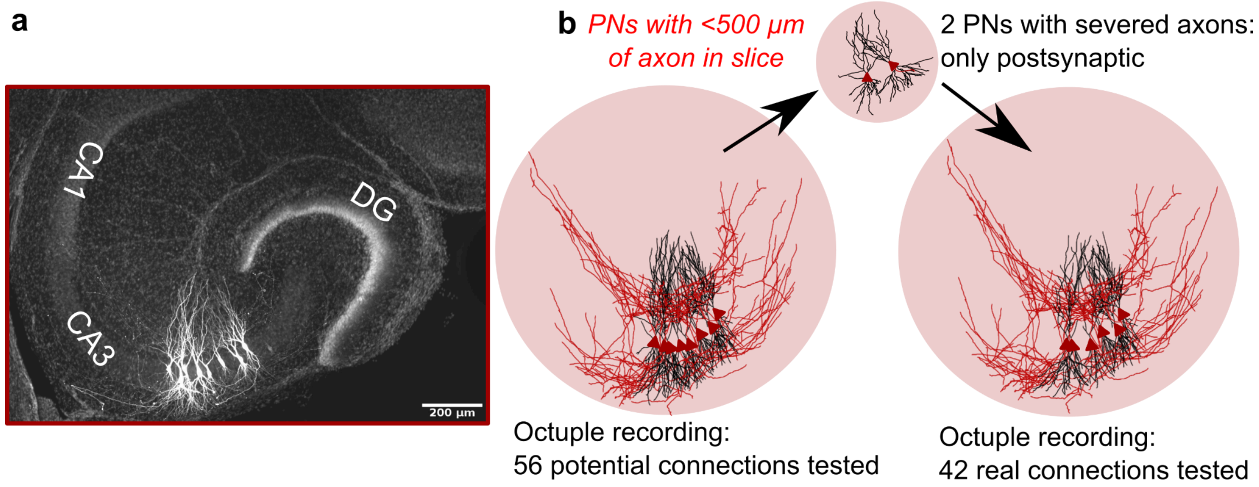

a Maximum intensity projection of a representative octuple recording from the P7−8 age group. **b** In this octuple, two PNs show < 500 µm of axon within the slice (severed during slicing procedure). These cells were therefore excluded as potential presynaptic partners. This procedure, applied to all DAB- and fluorescently-labeled PNs, allowed us to obtain a more accurate estimation of CA3−CA3 connection probability.

Supplementary Figure 4 | Within age group analysis reveals homogeneous properties of early postnatal, juvenile, and mature CA3 networks.

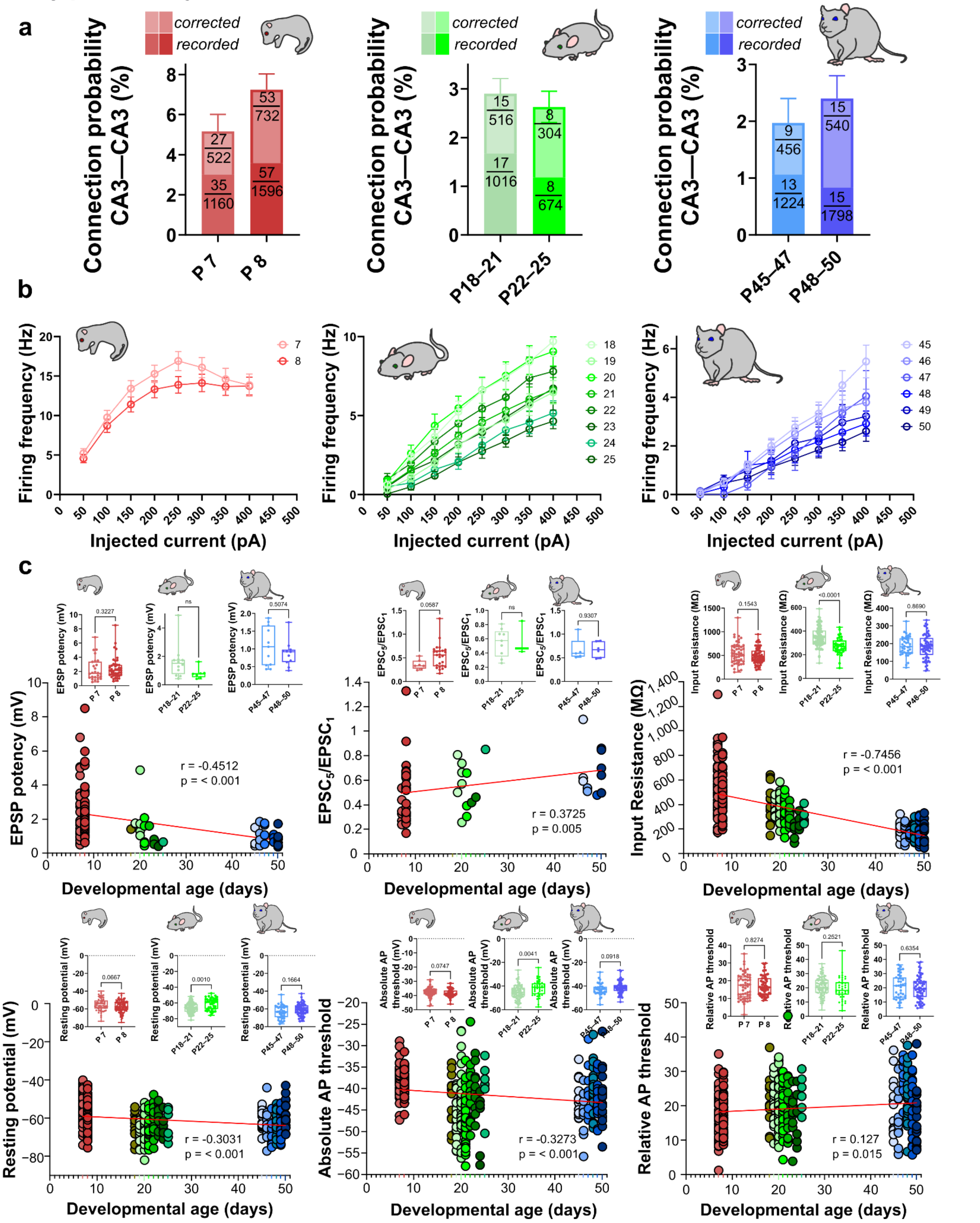

a Recorded and corrected values of connection probability of the early postnatal, juvenile, and mature CA3 networks were further analyzed by dividing each age group into smaller time bins. Statistical analysis revealed no differences in the probability of connection for CA3 PNs between

these subcategories (two-sided Fisher's exact test). **b** The relationship of injected current vs. firing frequency was further analyzed and the data of each age group were sorted per day within each category. Inset shows box plots (points, individual data; whiskers, min. to max.; box, 25–75% values; horizontal line, median) of firing frequency at 400 pA of injected current for PNs of animals of the same age. Reported *p* values obtained from non-parametric Kruskal-Wallis test, followed by two-sided Dunn's multiple comparisons test (for the comparisons within P18–25 and P45–50, only *p* values < 0.1 are shown). **c** Correlation (Spearman's rank) analysis of EPSP potency, $EPSC_5$ / $EPSC_1$, input resistance, resting potential, absolute AP threshold, and relative AP threshold against the entire developmental time period studied. Although every parameter measured showed a significant positive or negative correlation, there were few cases in which there were statistically significant differences between subcategories of each age group (e.g., input resistance P18–21 vs. P22–25); insets show corresponding box plots. Reported *p* values obtained from non-parametric Kruskal-Wallis test, followed by two-sided Dunn's multiple comparisons test.

Supplementary Figure 5 | Distance-dependence of connection probability and subregion differences within CA3.

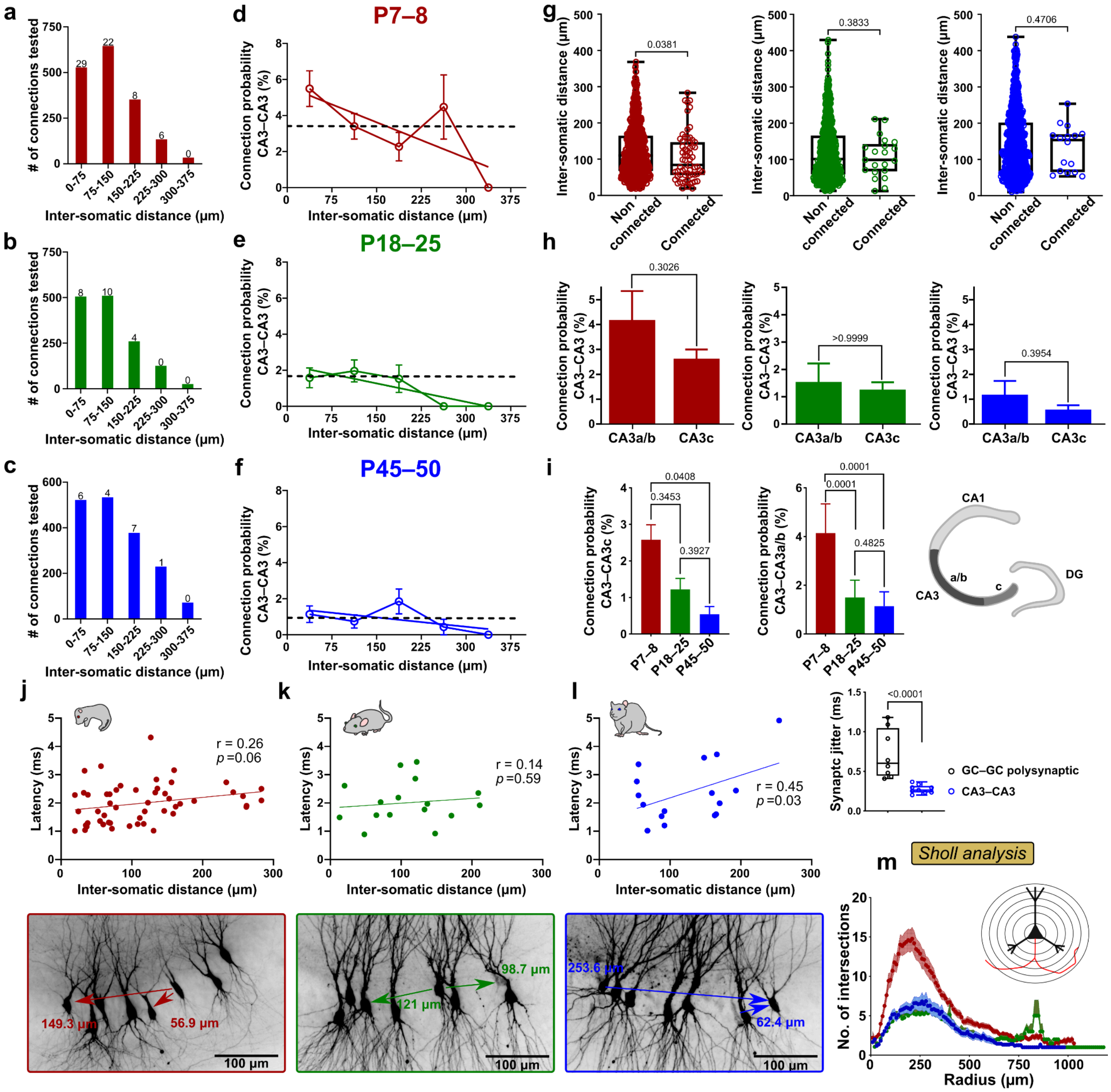

a–c Frequency histograms showing the number of tested connections per distance bin (75 μm) for the three developmental time points studied**.** The number of connections found per distance bin is shown above each plotted bar. **d–f** CA3–CA3 connection probability as a function of distance, for each age group. The horizontal dashed lines represent the global recorded connection probability. Error bars were calculated from a binomial distribution. Data were analyzed by linear regression. **g** Box plots (points, individual data; whiskers, min. to max.; box, 25–75% values; horizontal line, median) of inter-somatic distance of non-connected vs. connected PNs. Only the P7−8 connections showed a significant difference between non-connected and connected cells. Reported *p* values obtained from non-parametric Mann-Whitney test. **h** In all ages studied, there was a trend for a higher connection probability within CA3a/b (distal to the DG) than within CA3c (proximal to the DG; non-significant, two-sided

Fisher's exact test). Error bars calculated from a binomial distribution. **i** Connection probability comparison in CA3a/b and CA3c, across development. Reported *p* values obtained from Fisher's exact test. **j–l** Top, relation between latency and inter-somatic distance for connected pairs of PNs in each age group. Only in the mature P45–50 PN network, there was a statistically significant correlation between the two parameters (Spearman correlation: $r = 0.45$, $p = 0.03$). Bottom, maximum intensity projection micrographs of octuples, for all ages studied, showing Euclidean intersomatic distances of connected PNs. Synaptic jitter (SD of latency) of P45–50 EPSP latencies vs. GC–GC polysynaptic EPSPs are plotted as a confirmation of monosynaptic connections (longest latency jitter = 0.36 ms). Synaptic jitter: mean ± SEM; GC–GC polysynaptic: 0.71 ± 0.10 ms, $n = 8$; CA3–CA3: 0.27 ± 0.01 ms, $n = 9$. Reported *p* value obtained from non-parametric Mann-Whitney test. **m** Axonal Sholl analysis confirmed that P18–25 and P45–50 PNs have significantly less number of intersections per concentric ring (lesser degree of collateralization), with the larger differences corresponding to the inner rings (pruning confined to intra-CA3 collaterals). The latter suggests that the axonal pruning of PNs throughout development (**Fig. 3**) results in lower axon density.

Supplementary Figure 6 | Quantification of axonal subregion-targeting for each age group.

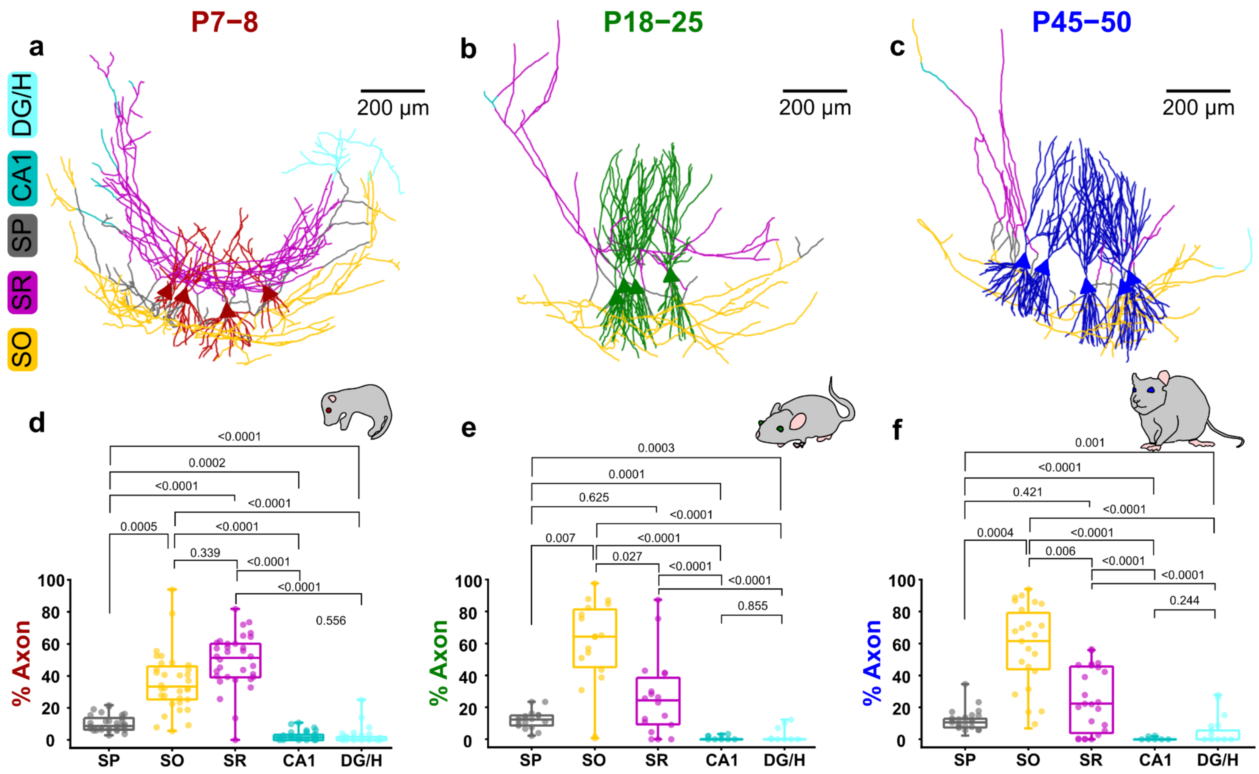

a–c Representative, segmented reconstructions of PNs for each developmental time period. For each reconstructed neuron, we measured the percentage of axon within CA3 *stratum pyramidale* (SP), *stratum oriens* (SO), *stratum radiatum* (SR), CA1, and dentate gyrus and / or hilus (DG/H). **d–f** Box plots (points, individual data; whiskers, min. to max.; box, 25–75% values; horizontal line, median) showing the percentage of axon that targets the specified layer / subregion. Axons belonging to PNs of early postnatal mice showed an even, stereotyped distribution in both SR and SO, whereas axons from juvenile and adult mice showed a progressive decline of the amount of axon targeted towards SR. For this analysis, we only included neurons whose axons were at least 1500 µm in length (P7–8, n = 34; P18–25, n = 18; P45–50, n = 24). Although much less abundant, PNs from the three developmental time points studied had axon collaterals back-projecting to the hilus or the dentate gyrus. Reported p values obtained from non-parametric Kruskal-Wallis test, followed by two-sided Dunn's multiple comparisons test.

Supplementary Figure 7 | Membrane properties of CA3 PNs show significant changes during postnatal development.

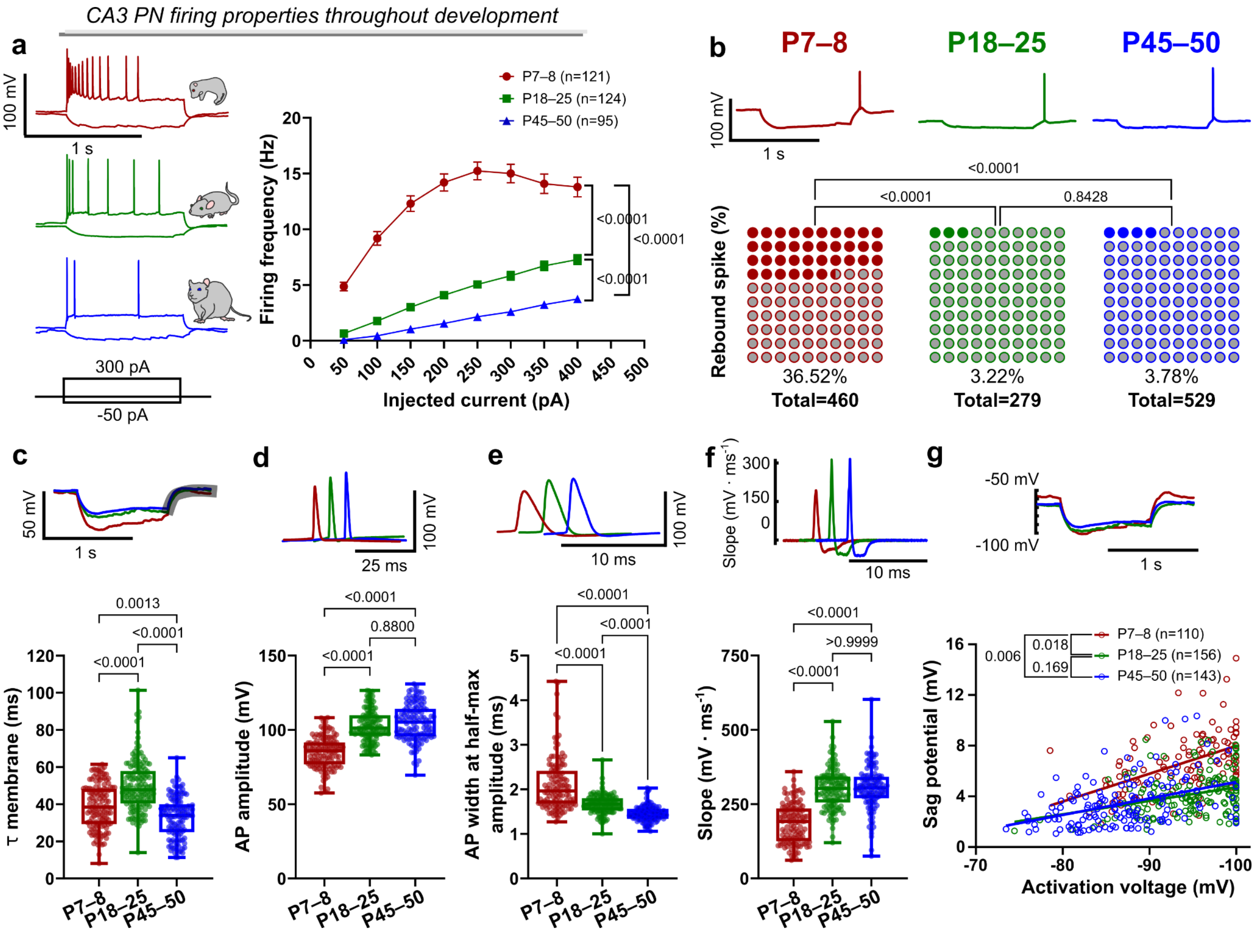

a Left, representative traces of the AP phenotype probed by 1-s long current injections for the three age groups. Right, average firing frequency vs. current relationships (f-I curves) of PNs from early postnatal, juvenile, and mature CA3 network. Data were analyzed by a mixed-effects model. **b** Percentage of neurons showing rebound APs (rebound spike %) across development. Reported *p* values obtained from two-sided Fisher's exact test. **c–f** Box plots (points, individual data; whiskers, min. to max.; box, 25–75% values; horizontal line, median) of values for apparent membrane time constant (**c**), AP amplitude (**d**), width of the AP at half-maximal amplitude (**e**), and dV/dt_{max} of AP (**f**). Apparent membrane time constant: mean ± SEM; P7−8: 38.78 ± 1.06 ms, $n = 128$; P18−25: 49.97 ± 1.12 ms, $n = 146$; P45−50: 32.95 ± 0.99 ms, $n = 122$. AP amplitude: mean ± SEM; P7−8: 84.82 ± 0.95 mV, $n = 122$; P18−25: 102.70 ± 0.95 mV, $n = 127$; P45−50: 104.80 ± 1.14 mV, $n = 118$. AP width at half-maximal amplitude: mean ± SEM; P7−8: 2.1 ± 0.05 ms, $n = 122$; P18−25: 1.66 ± 0.01 ms, $n = 130$; P45−50: 1.45 ± 1.01 ms, $n = 119$. dV/dt_{max}: mean ± SEM; P7−8: 186.3 ± 6.1 mV ms^{-1}, $n = 118$; P18−25: 302.70 ± 5.9 mV ms^{-1}, $n = 129$; P45−50: 304.1 ± 6.8 mV ms^{-1}, $n = 119$. Inset shows traces of the first derivative of the somatic membrane potentials. **g** Properties of sag upon hyperpolarizing current injection. Scatter plots with linear fits of sag potential vs. activation voltage, across development. Reported *p* values obtained from non-parametric Kruskal-Wallis test, followed by two-sided Dunn's multiple comparisons test.

Supplementary Figure 8 | Dendritic integration of convergent inputs.

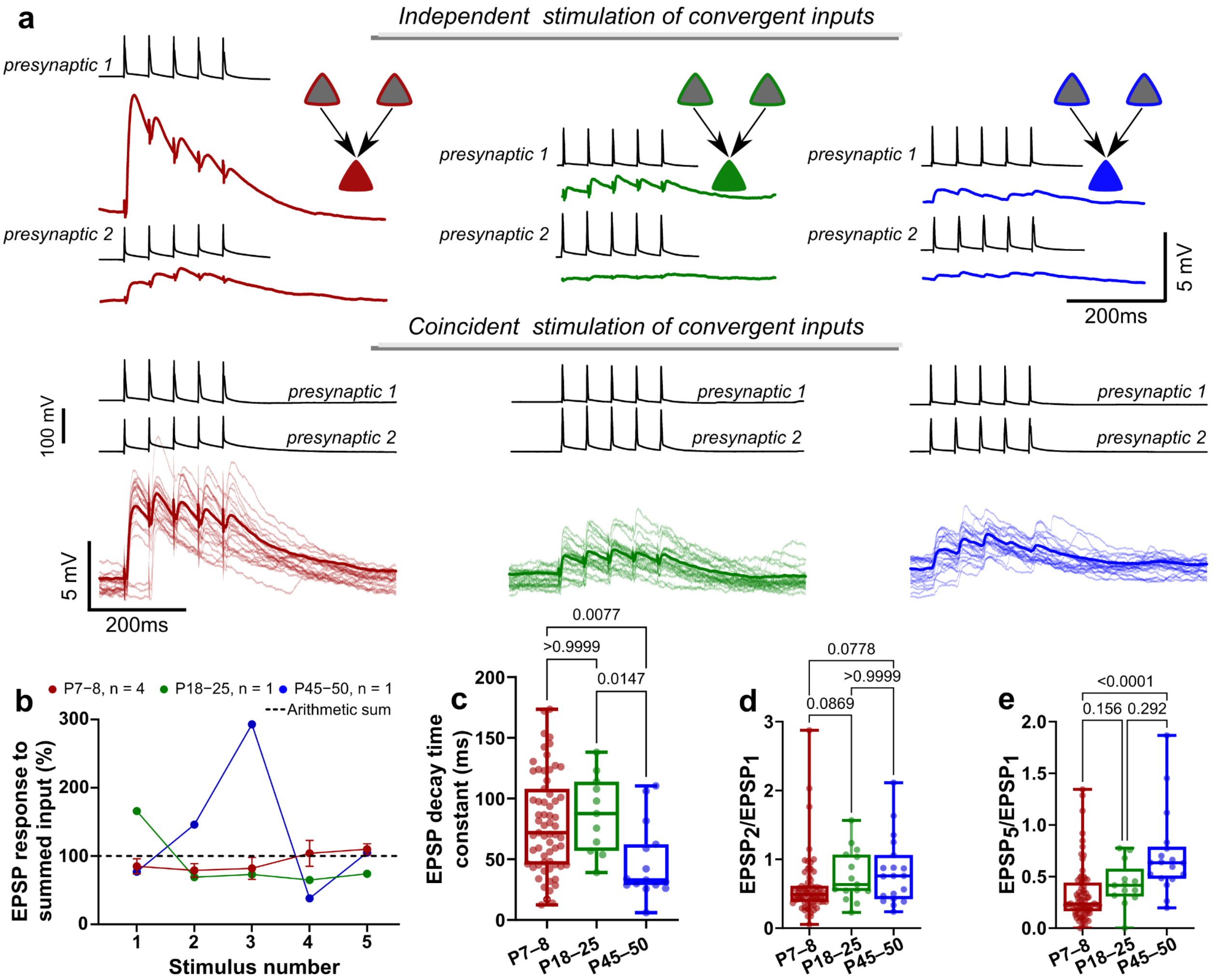

a Top: convergent presynaptic inputs across developmental time points were first independently stimulated and the evoked EPSPs in the postsynaptic PN were recorded. Bottom: both presynaptic inputs were stimulated simultaneously and the summed EPSP of both inputs was recorded in the corresponding postsynaptic PNs. **b** Normalized EPSP response to summed input for P7−8, P18−25, and P45−50 convergence motifs. Plotted circles represent recorded responses relative to each motif's arithmetic sum (dashed line); (4 convergence motifs in the P18–25 age group; 1 convergence motif at P18–25; 1 convergence motif at P45−50). **c–e** Box plots (points, individual data; whiskers, min. to max.; box, 25–75% values; horizontal line, median) of EPSP decay time constant (**c**), $EPSP_2$ / $EPSP_1$ (**d**), and $EPSP_5$ / $EPSP_1$ (**e**). EPSP decay time constant: mean ± SEM; P7−8: 79.66 ± 5.11 ms, n = 63; P18−25: 87.05 ± 9.61 ms, n = 11; P45−50: 46.97 ± 7.94 ms, n = 15. EPSP decay time constant significantly decreased with age. $EPSP_2$ / $EPSP_1$: mean ± SEM; P7−8: 0.58 ± 0.05, n = 70; P18−25: 0.76 ± 0.09, n = 15; P45−50: 0.79 ± 0.08, n = 22. $EPSP_5$ / $EPSP_1$: mean ± SEM; P7−8: 0.33 ± 0.03, n = 70; P18−25: 0.42 ± 0.05, n = 15; P45−50: 0.67 ± 0.07, n = 22. While paired-pulse ratio ($EPSP_2$ / $EPSP_1$) remained constant throughout development, $EPSP_5$ / $EPSP_1$ significantly increased with age, making P45−50 synapses less depressing. Reported p values obtained from non-parametric Kruskal-Wallis test, followed by two-sided Dunn's multiple comparisons test.

Supplementary Figure 9 | Quantitative analysis of pattern completion and pattern separation in the CA3 network.

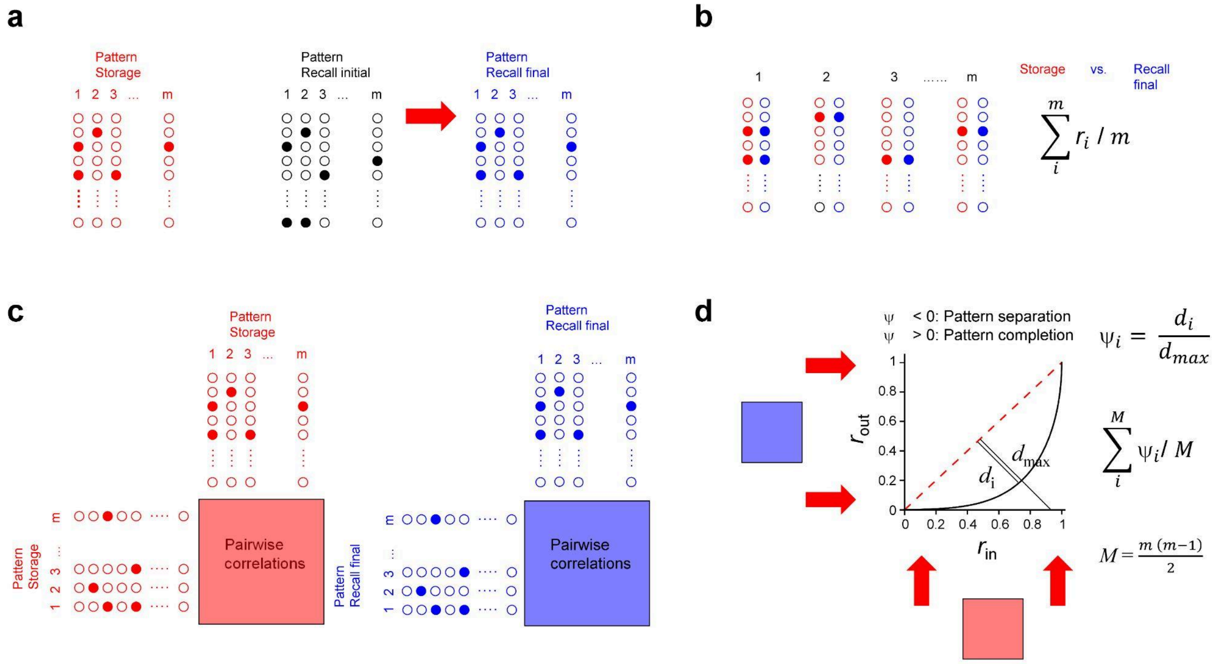

a Schematic illustration of patterns during storage (red), initial recall phase (black), and final recall phase (blue). Filled circles, active cells; open circles, inactive cells. **b** Analysis of successful recall and pattern completion based on comparison of stored patterns and final recall patterns. For m = 100 patterns, 100 pairs of vector comparisons are performed, and correlation coefficients (r_i; i = 1 .. 100) are averaged to determine pattern overlap. **c** Analysis of correlation structure in input and output patterns by pairwise comparison of initial storage patterns and corresponding final recall patterns. For m = 100 patterns, $M = m(m-1) / 2 = 4950$ pairwise comparisons are performed, and correlation coefficients (r_i; i = 1 .. 4950) are determined individually. **d** Analysis of pattern computations in input-output correlation graphs. Data points below the identity line (red dashed) indicate pattern separation, whereas data points above the identity line suggest pattern completion. Data points from the r_{in} correlation matrix are plotted on the abscissa, whereas the data points from the r_{out} correlation matrix are plotted on the ordinate. For each data point, a pattern similarity index (ψ_i) was computed from the perpendicular distance to the identity line. Finally, individual ψ_i values are averaged to give a global pattern separation index for the r_{out}–r_{in} graph.

Supplementary Figure 10 | Developmental changes of network parameters affect pattern computations.

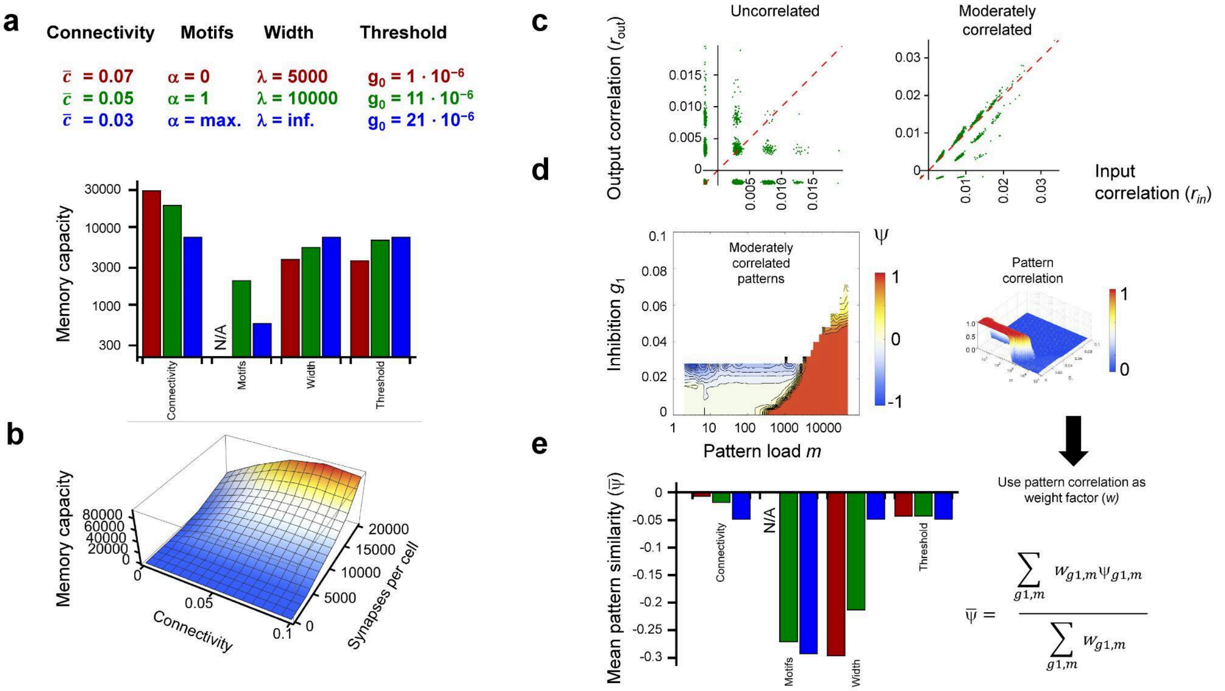

$$\overline{\psi} = \frac{\sum_{g1,m} w_{g1,m}\psi_{g1,m}}{\sum_{g1,m} w_{g1,m}}$$

a Analysis of memory capacity for changes in average connectivity ($\overline{c}$ = 0.07, 0.05, and 0.03), increasing abundance of nonrandom connectivity motifs (random connectivity, all α values = 1, and all α values near maximum – $\overline{c}$ = 0.02, α_{recip} = 5, α_{conv} = 5, α_{div} = 5, and α_{chain} = 3), width of connectivity (as implemented by λ = 5000 cells, 10,000 cells, and lack of distance dependence, with c_{peak} = 0.3, 0.15, and 0.03, respectively, to preserve average total connectivity), and number of converging inputs required to initiate spikes in single cells (as implemented via parameter g_0 = 1×10^{-6}, 11×10^{-6}, and 21×10^{-6}). Bars correspond to *C* values shown in Fig. 7b–e. **b** Plot of memory capacity against connectivity and number of synapses per cell. Note that memory capacity markedly changes if synapse number is freely changing (see a), but less so if the number of synapses per cell are kept constant. **c** Output-input (r_{out}–r_{in}) correlation plots. Pairwise pattern correlations were determined for $\overline{c}$ = 0.05 and g_0 = 21×10^{-6}; network parameters were default values otherwise (**Supplementary Table 5**). ψ was computed from pairwise correlations of 100 patterns, comparing final patterns after retrieval (10 iterations) with original patterns used for storage. Left, r_{out}–r_{in} plot in which input patterns were uncorrelated; right, plot in which input patterns were moderately correlated. Red dashed lines, identity lines. **d** Plot of pattern similarity index (ψ) against pattern load (m) and inhibition factor (g_1) for $\overline{c}$ = 0.05 and g_0 = 21×10^{-6}. To probe pattern separation, moderately correlated initial patterns were used in these simulations. Inset shows corresponding pattern correlation plot (memory capacity slightly smaller than in Fig. 7a, center, because moderately correlated patterns were used). **e** Mean ψ values for the same network parameters as shown in a. Mean ψ values were computed as the weighted mean of ψ over the m–g_1 parameter space, using corresponding memory capacity values as weight factors. Reducing connectivity, introducing motifs, and increasing activation threshold reduced ψ (i.e. shifted in the direction of pattern separation), whereas increasing broadness of connectivity increased it (i.e. shifted in the direction of pattern completion). Red-green-blue color code represents the direction of developmental changes in the experimental data.

Supplementary Table 1. Active and passive membrane properties of CA3 PNs over postnatal development.

Parameter	P7–8	P18–25	P45–50	*p* values[1]
Membrane resistance (MΩ)	505.6 ± 17.0 [147.7–947.3] *n* = 116	338 ± 7.2 [129.9–587.5] *n* = 123	183.8 ± 6.6 [47.1–332.6] *n* = 91	<0.0001 (<0.0001; <0.0001; <0.0001)
Resting potential (mV)	−56.72 ± 0.5 [−75.3– −40.0] *n* = 166	−65.2 ± 0.2 [−85.5– −47.6] *n* = 123	−61.08 ± 0.69 [−73.9– −42.7] *n* = 91	<0.0001 (<0.0001; <0.0001; 0.0005)
Absolute AP threshold (mV)	−40.76 ± 0.55 [−47.2– −28.9] *n* = 40	−44.7 ± 0.6 [−58– −24.35] *n* = 123	−41.3 ± 0.5 [−53.5–−26.6] *n* = 89	<0.0001 (<0.0001; >0.9999; <0.0001)
Relative AP threshold (mV)	20.67 ± 0.6 [10.7–32.6] *n* = 40	20.6 ± 0.6 [4.3–46.2] *n* = 123	19.7 ± 0.9 [14.3–23.6] *n* = 89	0.0009 (0.0007; 0.0729; 0.7683)
Apparent membrane time constant (ms)	38.78 ± 1.06 [8.12–53.28] *n* = 128	49.97 ± 1.12 [13.97–101.3] *n* = 146	32.95 ± 9.99 [11.35–64.95] *n* = 122	<0.0001 (<0.0001; 0.0013; <0.0001)
AP amplitude (mV)	84.8 ± 0.95 [57.6–108.2] *n* = 122	102.7 ± 0.95 [83.15–126.5] *n* = 127	104.8 ± 1.14 [57.7–130.9] *n* = 118	<0.0001 (<0.0001; <0.0001; 0.8800)
Width of AP at half-maximum amplitude (ms)	2.11 ± 0.60 [1.3–4.4] *n* = 122	1.66 ± 0.21 [1–2.7] *n* = 130	1.45 ± 0.18 [1.1–2] *n* = 119	<0.0001 (<0.0001; <0.0001; <0.0001)
dV/dt_{max} ($mV\ ms^{-1}$)	186.3 ± 6.1 [61.4–297.6] *n* = 118	302.7 ± 5.9 [120.1–407.8] *n* = 129	304.1 ± 6.8 [75.2–527] *n* = 118	<0.0001 (<0.0001; <0.0001; >0.9999)

Values are given as mean ± SEM [range], with *n* indicating the number of recorded cells.

1 *p* values indicate results from Kruskal-Wallis test (outside parentheses) and two-sided Dunn's multiple-comparison tests (inside parentheses). The order of pairwise comparisons is P7–8 vs. P18–25; P18–25 vs. P45–50; and P7–8 vs. P45–50.

Supplementary Table 2. Voltage-clamp analysis of CA3–CA3 synapses over postnatal development.

Parameter	P7–8	P18–25	P45–50	*p* values[1]
1st EPSC amplitude (pA)	27.51 ± 4.01 [7.40–51.54] *n* = 15	31.74 ± 6,83 [11.08–51.56] *n* = 7	17.19 ± 2.78 [4.86–37.22] *n* = 11	0.1550 (>0.9999; 0.2845; 0.3002)
1st EPSC potency (pA)	36.16 ± 3.77 [14.78–64.4] *n* = 15	37.31 ± 6.04 [15.76–60.97] *n* = 7	23.36 ± 2.82 [8.79–42.32] *n* = 11	0.0606 (>0.9999; 0.0802; 0.2222)
1st EPSC 20–80% rise time (ms)	1.06 ± 0.07 [0.58–1.65] *n* = 15	1.38 ± 0.19 [0.9–2.37] *n* = 7	1.35 ± 0.10 [0.88–1.91] *n* = 11	0.1315 (0.5678; 0.1711; >0.9999)
1st EPSC latency (ms)	1.99 ± 0.08 [1.01–4.32] *n* = 60	1.90 ± 0.15 [0.92–3.27] *n* = 17	2.69 ± 0.28 [1.2–5.39] *n* = 18	0.0480 (>0.9999; 0.0741; 0.0887)
1st EPSC decay time constant (ms)	7.04 ± 0.63 [3.72–7.27] *n* = 15	11.94 ±1.47 [5.4–17.24] *n* = 7	9.81 ± 1.01 [3.83–14.7] *n* = 11	0.0102 (0.0160; 0.1011; >0.9999)
Proportion of failures	0.25 ± 0.02 [0–0.85] *n* = 57	0.3 ± 0.06 [0–0.73] *n* = 17	0.4 ± 0.05 [0.06–0.81] *n* = 16	0.0746 (>0.9999; 0.0681; 0.4486)
PPR	0.7 ± 0.06 [0.25–1.97] *n* = 32	0.72 ± 0.05 [0.58–0.94] *n* = 7	0.76 ± 0.05 [0.43–1.08] *n* = 12	0.3672 (>0.9999; 0.4923; >0.9999)
$EPSC_5$ / $EPSC_1$	0.49 ± 0.04 [0.17–1.33] *n* = 32	0.5 ± 0.08 [0.25–0.85] *n* = 7	0.62 ± 0.03 [0.49–0.85] *n* = 10	0.1319 (>0.9999; 0.1405; 0.5084)
Series resistance (MΩ)	12.81 ± 0.8 [7.76–17.76] *n* = 16	12.66 ± 2.24 [8.12–19.51] *n* = 7	10.44 ± 1.3 [5.94–19] *n* = 10	0.2907 (>0.9999; 0.3629; >0.9999)

Values are given as mean ± SEM [range], with *n* indicating the number of recorded synaptically connected pairs.

1 *p* values indicate results from Kruskal-Wallis test (outside parentheses) and two-sided Dunn's multiple-comparison tests (inside parentheses). The order of pairwise comparisons is P7–8 vs. P18–25; P18–25 vs. P45–50; and P7–8 vs. P45–50.

Supplementary Table 3. Current-clamp analysis of CA3–CA3 synapses over postnatal development.

Parameter	P7–8	P18–25	P45–50	*p* values[1]
1st EPSP amplitude (mV)	1.94 ± 0.18 [0.17–8.51] *n* = 70	1.34 ± 0.27 [0.15–4.9] *n* = 16	0.59 ± 0.08 [0.05–1.15] *n* = 25	<0.0001 (0.7128; <0.0001; 0.0234)
1st EPSP potency (mV)	2.39 ± 0.18 [0.52–8.51] *n* = 70	1.43 ± 0.26 [0.43–4.89] *n* = 16	0.9 ± 0.09 [0.31–1.87] *n* = 25	<0.0001 (0.0155; <0.0001; 0.2908)
1st EPSP decay time constant (ms)	79.66 ± 5.11 [12.4–173.4] *n* = 63	87.05 ± 9.61 [39.0–138.1] *n* = 11	46.97 ± 7.94 [5.98–110.3] *n* = 15	0.0046 (>0.9999; 0.0077; 0.0147)
PPR	0.58 ± 0.05 [0.05–2.87] *n* = 70	0.76 ± 0.09 [0.22–1.56] *n* = 15	0.79 ± 0.07 [0.23–2.11] *n* = 22	0.0229 (0.0883; 0.1122; >0.9999)
$EPSP_5$ / $EPSP_1$	0.33 ± 0.03 [0.2–1.34] *n* = 70	0.42 ± 0.05 [0–0.77] *n* = 13	0.67 ± 0.09 [0.19–1.86] *n* = 20	<0.0001 (0.1520; <0.0001; 0.4786)
Relative AP threshold / EPSP	7.03	15.37	24.70	N/A

Values are given as mean ± SEM [range], with *n* indicating the number of recorded synaptically connected pairs. N/A, not applicable.

1 *p* values indicate results from Kruskal-Wallis test (outside parentheses) and two-sided Dunn's multiple-comparison tests (inside parentheses). The order of pairwise comparisons is P7–8 vs. P18–25; P18–25 vs. P45–50; and P7–8 vs. P45–50.

Supplementary Table 4. Morphological measurements of CA3 PNs throughout development.

Parameter	P7–8	P18–25	P45–50	*p* values[1]
Cumulative axonal length (mm)	8.13 ± 0.64 mm<br>[20.31–1.44]<br>*n* = 35	3.69 ± 0.49<br>[9.54–1.01]<br>*n* = 21	3.7 ± 0.45<br>[9.16–1.1]<br>*n* = 29	<0.0001 (0.0001; >0.9999; 0.0001)
Number of axonal branch points	83.6 ± 6.39<br>[200–13]<br>*n* = 35	24.14 ± 2.98<br>[56–5]<br>*n* = 21	24.00 ± 3.42<br>[93–3]<br>*n* = 29	<0.0001 (0.0001; >0.9999; 0.0001)
Apical dendritic length (μm)	1.12 ± 0.9<br>[3.4–0.44]<br>*n* = 39	3.60 ± 0.25<br>[6.04–1.61]<br>*n* = 28	3.70 ± 0.25<br>[8.48–0.98]<br>*n* = 38	<0.0001 (0.0001; >0.9999; 0.0001)
Basal dendritic length (μm)	1.06 ± 0.9<br>[2.48–0.45]<br>*n* = 39	1.8 ± 0.13<br>[3.76–0.86]<br>*n* = 28	1.80 ± 0.15<br>[3.9–0.32]<br>*n* = 38	<0.0001 (0.0002; >0.9999; 0.0007)
Spine density SR (μm^{-1})	0.30 ± 0.02<br>[0.68–0.1]<br>*n* = 24	0.83 ± 0.03 μm<br>[1.43–0.44]<br>*n* = 24	0.90 ± 0.05<br>[1.35–0.43]<br>*n* = 25	<0.0001 (0.0001; >0.6355; 0.0001)
Spine density SO (μm^{-1})	0.2 ± 0.02<br>[0.42–0.03]<br>*n* = 23	0.64 ± 0.02<br>[0.93–0.44]<br>*n* = 23	0.80 ± 0.02<br>[1.11–0.36]<br>*n* = 25	<0.0001 (0.0001; >0.1521; 0.0001)
Dendrite diameter (μm)	1.8 ± 0.04<br>[2.75–1.05]<br>*n* = 76	1.98 ± 0.04<br>[3.36–1.08]<br>*n* = 77	2.51 ± 0.06<br>[4.38–1.28]<br>*n* = 77	<0.0001 (0.0971; 0.0001; 0.0001)
Axonal target space SR (cm^3)	6364.94	9947.35	10562.14	N/A
Axonal target space SO (cm^3)	3097.16	4990.96	5804.48	N/A

Values are given as mean ± SEM [range], with *n* indicating the number of reconstructed neurons for axonal and dendritic length, the number of individual neurons in which spines were counted for spine densities, and the number of dendritic branches measured for dendritic diameter. Axonal target space is the product of slice thickness, average layer length, and average CA3 proximo-distal axis length.

1 *p* values indicate results from Kruskal-Wallis test (outside parentheses) and Dunn's multiple-comparison tests (inside parentheses). The order of pairwise comparisons is P7–8 vs. P18–25; P18–25 vs. P45–50; and P7–8 vs. P45–50.

Supplementary Table 5. Parameters of the autoassociative network model of pattern completion.

**Parameter**	**Explanation**	**Default value or range**	**Alternative values or range (if indicated)**
n	Number of neurons	100,000	50,000–500,000
$\overline{c}$	Mean connection probability	3%	2%, 5%, 7%
f	Mean activity level	0.002	0.001, 0.003
g_0	Firing threshold	21×10^{-6}	1×10^{-6}, 11×10^{-6}
g_1	Inhibition factor	0 – 0.1	
m	Pattern load (number of patterns applied in storage phase)	0 – 100,000	Up to 1,000,000
b_{valid}	Proportion of valid firings in initial phase of recall (b_{valid} = 1 → identity to initial pattern)	0.5	
$b_{spurious}$	Proportion of spurious firings in initial phase of recall ($b_{spurious}$ = 0 → no spurious firing)	0.001	
α_{recip}	Abundance of reciprocal motifs in comparison to random network	0 (random)	1 – 5
α_{conv}	Abundance of convergence motifs	0 (random)	1 – 5
α_{div}	Abundance of divergence motifs	0 (random)	1 – 5
α_{chain}	Abundance of disynaptic chain motifs	0 (random)	1 – 3
λ	Length constant of distance dependence	Lack of distance dependence (λ = infinity)	Local connectivity (λ = 5000 or 10,000 cells)
c_{peak}	Peak connection probability	0.03	0.15, 0.3
