# Supplementary material for: Developmental emergence of sparse and structured synaptic connectivity in the hippocampal CA3 memory circuit
Source: Nat Commun. 2026 Apr 21;17:5540. doi: 10.1038/s41467-026-71914-x (PMC13287488; doi:10.1038/s41467-026-71914-x)
Supplement: Supplementary file 2 — Reporting Summary [file 41467_2026_71914_MOESM2_ESM.pdf]

nature portfolio

Corresponding author(s): Peter Jonas

Last updated by author(s): Mar 17, 2026

Reporting Summary

Nature Portfolio wishes to improve the reproducibility of the work that we publish. This form provides structure for consistency and transparency in reporting. For further information on Nature Portfolio policies, see our Editorial Policies and the Editorial Policy Checklist.

Statistics

For all statistical analyses, confirm that the following items are present in the figure legend, table legend, main text, or Methods section.

n/a	Confirmed	
☐	☒	The exact sample size (*n*) for each experimental group/condition, given as a discrete number and unit of measurement
☐	☒	A statement on whether measurements were taken from distinct samples or whether the same sample was measured repeatedly
☐	☒	The statistical test(s) used AND whether they are one- or two-sided<br>*Only common tests should be described solely by name; describe more complex techniques in the Methods section.*
☒	☐	A description of all covariates tested
☐	☒	A description of any assumptions or corrections, such as tests of normality and adjustment for multiple comparisons
☐	☒	A full description of the statistical parameters including central tendency (e.g. means) or other basic estimates (e.g. regression coefficient) AND variation (e.g. standard deviation) or associated estimates of uncertainty (e.g. confidence intervals)
☐	☒	For null hypothesis testing, the test statistic (e.g. *F*, *t*, *r*) with confidence intervals, effect sizes, degrees of freedom and *P* value noted<br>*Give P values as exact values whenever suitable.*
☒	☐	For Bayesian analysis, information on the choice of priors and Markov chain Monte Carlo settings
☒	☐	For hierarchical and complex designs, identification of the appropriate level for tests and full reporting of outcomes
☒	☐	Estimates of effect sizes (e.g. Cohen's *d*, Pearson's *r*), indicating how they were calculated

Our web collection on statistics for biologists contains articles on many of the points above.

Software and code

Policy information about availability of computer code

Data collection	Recordings were performed using 4 700B Multiclamp amplifiers (Molecular Devices). Signals were digitized at 20 kHz with a CED 1401 power3 AD/DA converter (Cambridge Electronic Design). Pulse generation and data acquisition were performed using Signal software 6.0 (Cambridge Electronic Design). Imaging was done using an Olympus BX61 wide-field and an Andor Dragonfly confocal microscope (dual spinning disk with 25 and 40 µm pinholes, Oxford Instruments).
Data analysis	Data was analyzed using either Stimfit or equivalent Python-based scripts, as well as Matlab- (Mathworks; MATLAB R2024a) scripts. Neurons were reconstructed using Neutube Build Version 1.0z (2018.07.12), which is a Fiji plugin. Plots were generated using GraphPad Prism 10.2.2 and figures were rendered using Inkskape.

For manuscripts utilizing custom algorithms or software that are central to the research but not yet described in published literature, software must be made available to editors and reviewers. We strongly encourage code deposition in a community repository (e.g. GitHub). See the Nature Portfolio guidelines for submitting code & software for further information.

Data

Policy information about availability of data

All manuscripts must include a data availability statement. This statement should provide the following information, where applicable:

- Accession codes, unique identifiers, or web links for publicly available datasets
- A description of any restrictions on data availability
- For clinical datasets or third party data, please ensure that the statement adheres to our policy

Source data are provided with this paper. Additional original data are available from the corresponding author upon request.

Research involving human participants, their data, or biological material

Policy information about studies with human participants or human data. See also policy information about sex, gender (identity/presentation), and sexual orientation and race, ethnicity and racism.

Reporting on sex and gender	N/A
Reporting on race, ethnicity, or other socially relevant groupings	N/A
Population characteristics	N/A
Recruitment	N/A
Ethics oversight	N/A

Note that full information on the approval of the study protocol must also be provided in the manuscript.

Field-specific reporting

Please select the one below that is the best fit for your research. If you are not sure, read the appropriate sections before making your selection.

☒ Life sciences ☐ Behavioural & social sciences ☐ Ecological, evolutionary & environmental sciences

For a reference copy of the document with all sections, see nature.com/documents/nr-reporting-summary-flat.pdf

Life sciences study design

All studies must disclose on these points even when the disclosure is negative.

Sample size	No statistical method was used to predetermine sample sizes. For all age groups, we report the number of recordings performed, including the number of cells recorded in each experiment and the total number of potential connections tested, including a correction method to estimate a more accurate connection probability.
Data exclusions	No data were excluded from the analysis.
Replication	Reproducibility trials were conducted along the study by other scientists from the same lab using a different setup, with no statistical differences in the obtained results. We also conducted interleaved experiments using all three age groups in different seasons of the year, with no apparent differences.
Randomization	No randomization was implemented.
Blinding	Blinding of the researchers in relation to the age group was not implemented.

Reporting for specific materials, systems and methods

We require information from authors about some types of materials, experimental systems and methods used in many studies. Here, indicate whether each material, system or method listed is relevant to your study. If you are not sure if a list item applies to your research, read the appropriate section before selecting a response.

Materials & experimental systems

n/a	Involved in the study
☒	☐ Antibodies
☒	☐ Eukaryotic cell lines
☒	☐ Palaeontology and archaeology
☐	☒ Animals and other organisms
☒	☐ Clinical data
☒	☐ Dual use research of concern
☒	☐ Plants

Methods

n/a	Involved in the study
☒	☐ ChIP-seq
☒	☐ Flow cytometry
☒	☐ MRI-based neuroimaging

Animals and other research organisms

Policy information about studies involving animals; ARRIVE guidelines recommended for reporting animal research, and Sex and Gender in Research

Laboratory animals	We obtained data from wild type C57BL/6J laboratory mice. We compared data from animals at three different developmental time-points, corresponding to early postnatal (P7–8), juvenile (P18–25), and young adult (P45–50).
Wild animals	No wild animals were used.
Reporting on sex	Both sexes were used.
Field-collected samples	No field-collected samples were used in this study.
Ethics oversight	All procedures were performed in strict accordance with institutional, national, and European guidelines for animal experimentation, approved by the Bundesministerium für Bildung, Wissenschaft und Forschung of Austria.

Note that full information on the approval of the study protocol must also be provided in the manuscript.

Plants

Seed stocks	*Report on the source of all seed stocks or other plant material used. If applicable, state the seed stock centre and catalogue number. If plant specimens were collected from the field, describe the collection location, date and sampling procedures.*
Novel plant genotypes	*Describe the methods by which all novel plant genotypes were produced. This includes those generated by transgenic approaches, gene editing, chemical/radiation-based mutagenesis and hybridization. For transgenic lines, describe the transformation method, the number of independent lines analyzed and the generation upon which experiments were performed. For gene-edited lines, describe the editor used, the endogenous sequence targeted for editing, the targeting guide RNA sequence (if applicable) and how the editor was applied.*
Authentication	*Describe any authentication procedures for each seed stock used or novel genotype generated. Describe any experiments used to assess the effect of a mutation and, where applicable, how potential secondary effects (e.g. second site T-DNA insertions, mosiacism, off-target gene editing) were examined.*
