# Supplementary material for: Developmental emergence of sparse and structured synaptic connectivity in the hippocampal CA3 memory circuit
Source: Nat Commun. 2026 Apr 21;17:5540. doi: 10.1038/s41467-026-71914-x (PMC13287488; doi:10.1038/s41467-026-71914-x)
Supplement: Supplementary file 3 — Transparent Peer Review File [file 41467_2026_71914_MOESM3_ESM.pdf]

nature portfolio

Peer Review File

Developmental emergence of sparse and structured synaptic connectivity in the hippocampal CA3 memory circuit

Corresponding Author: Professor Peter Jonas

This file contains all reviewer reports in order by version, followed by all author rebuttals in order by version.

Version 0:

Reviewer comments:

Reviewer #1

(Remarks to the Author)
In this paper, the authors performed simultaneous patch-clamp recordings from multiple CA3 pyramidal neurons in acute rodent brain slices at different developmental time points. They rigorously characterized morphological and functional features of the neurons and synaptic connections, and related changes in function to memory capacity with modeling. The scope of the study is very focused, the data is high-quality, and the conclusions are well-supported by the data. Below are suggestions to improve the manuscript.

P3: Here are additional relevant citations for connectivity structured by birth date:
• Druckmann S, Feng L, Lee B, Yook C, Zhao T, Magee JC, Kim J. Structured synaptic connectivity between hippocampal regions. Neuron. 2014 Feb 5;81(3):629-40.
• Huszár, R., Zhang, Y., Blockus, H. et al. Preconfigured dynamics in the hippocampus are guided by embryonic birthdate and rate of neurogenesis. Nat Neurosci 25, 1201–1212 (2022).

P30, Fig 2a: Expressing the numbers of connections in these bar plots as found over tested would be more intuitive.

P30, Fig. 2b,c: These panels are not discussed in the text. The equation under Fig. 2b is not discussed in the figure legend.

P34, Fig. 5a: Extra space between “Unitary” and “EPSP” in panel subtitle.

P34, Fig. 5d: Inset is not described in legend.

P35, Fig. 6e: I find the clipped spikes and the upward / downward arrows are not helping with understanding the figure. Perhaps just use two shaded bars of color to indicate where presynaptic stimulation vs postsynaptic current injection occurred, and show the full spike height in both cases.

P8:
• “synapses in the P7–8 age group were significantly more depressing.” Perhaps this should reference Supp. Fig. 7e (P47) where you quantify short term adaptation of EPSP amplitude.
• “Storage of patterns was accomplished via a clipped Hebbian synaptic plasticity rule.” Perhaps this should reference a Methods section or a Supp. Fig. that concisely explains this rule.

P9:
• “decreased memory capacity, as the total number of synapses became reduced.” But didn’t the data show increased dendrite length and spine density? Is it possible in this model to separately control connection probability and number of synapses?
• Given that different developmentally-regulated network parameters either increase or decrease storage capacity, is there a final conclusion about whether we expect the net difference to be an increase or a decrease by adulthood?

P21: The provided explanation of the method to quantify pattern separation by a difference between input-input correlations and output-output correlations is insufficient. Please expand for clarity. It would be even better to provide a supplementary figure showing the scatter plots and illustrating the method. Also, a Guzman et al., 2021 is cited in line, but does not appear

in the References. It is likely a typo referring to Guzman et al., 2016. That study does not completely explain the method either, and I couldn't find it along the citation chain from there either.

Reviewer #2

(Remarks to the Author)
The recurrent connections in the hippocampal CA3 region are considered to be a key neural mechanism underlying pattern completion and memory retrieval. This study investigates how these recurrent connections in CA3 develop across different stages. The work provides important insights into infant learning and memory, as well as the function of CA3 in adulthood. The authors show that at P7–8, CA3 recurrent connectivity is relatively dense and synaptic weights are relatively strong. With development, at P18–25 and P45–50, both connection probability and synaptic strength decline. Computational modeling further demonstrates that these sparse recurrent connections can effectively support memory storage, retrieval, and pattern completion. Their findings also suggest that the emergence of the adult-like connectivity pattern in CA3, i.e., the disynaptic connectivity motif, is shaped by experience-dependent processes, rather than being entirely preconfigured. Overall, this study represents an important advance in our understanding of the development of the hippocampus and memory. I have some comments that should be addressed before publication:

1. About the choice of developmental stage groups: while P7–8 and P45–50 are appropriate for representing early postnatal and near-adult stages, respectively, the P18–25 interval spans a relatively long developmental period that encompasses several important milestones: grid cells emerge around P20–21, infantile amnesia exists at P17–18 and disappears at P24, and memory precision appears by ~P24 (https://pubmed.ncbi.nlm.nih.gov/40215964/). If feasible, it would be informative to subdivide this window into P18–20 and P23–25 (or use another reasonable subdivision) and assess at least one key metric (e.g., connection probability, synaptic strength, or spine density). Such a subdivision could help determine whether the observed developmental trend is genuinely gradual or instead reflects a more discrete transition. If the authors' intention is simply to demonstrate a monotonic, gradual refinement across P7–8, P18–25, and P45–50, rather than to emphasize a specific developmental period within P18–25, the use of the broader window is acceptable, but it should be explicitly acknowledged.

2. Relatedly, the text states: "episodic memory develops relatively late, approximately two years after birth. As this time point in humans is thought to correspond to the developmental range P14–21 in mice (Bevandić et al., 2024), these changes may be related to the developmental phenomena investigated in the present paper". This description is not entirely accurate. In rodents, the emergence of precise episodic-like memory formation is generally reported around P24, rather than P14–21 (https://pubmed.ncbi.nlm.nih.gov/40215964/).

3. Regarding the model, the parameter "pattern similarity index" might require clarification with a schematic illustration: what are its precise inputs and outputs? In the text, the network input is described as "decorrelated information from the dentate gyrus". Although the mossy-fiber tract length appears unchanged across development, as shown in this study, its density may vary as the number of granule cells increases; consequently, the inputs to CA3 could differ across developmental stages.

4. In addition, regarding the model, the experimental results in this study have shown a trend for recurrent connectivity to be higher in CA3a/b than in CA3c, supporting the view that CA3a/b favors pattern completion while CA3c supports pattern separation (see https://pubmed.ncbi.nlm.nih.gov/26298277/ and https://pubmed.ncbi.nlm.nih.gov/26298276/). The present study reported a decrease in connection probability across these subregions with development. Why does the model predict that both pattern completion and pattern separation increase with development across CA3 subregions?

5. The authors propose that the observed connectivity pattern in CA3 is sculpted by experience rather than predetermined, noting that early in development, connectivity is relatively dense, whereas disynaptic motifs are rare, compared to later developmental stages. However, an alternative possibility is that the connectivity observed at P7–8 is largely predetermined, and that the motifs seen later simply reflect those that remain after developmental elimination—arising from a combination of both innate programs and experience, rather than experience alone. If experience were the sole contributor, one would expect these motifs to be formed de novo rather than selectively eliminated from an initially dense network. The authors might therefore consider discussing how to distinguish between selective elimination and de novo formation (for example, by longitudinal imaging).

6. This study demonstrated a developmental decrease in connection probability, a pruning of short-distance recurrent synapses, and a decrease in transmission efficacy. Some mechanistic speculation should be provided. One plausible contributor is the maturation of inhibition. Parvalbumin-positive (PV) interneurons in CA3, as in other hippocampal subfields, undergo significant maturation during development (https://pubmed.ncbi.nlm.nih.gov/28154241/).

7. The authors report a developmental decline of "detonating" (high-fidelity, single-spike-driven) synaptic transmission in CA3. A similar phenomenon has been observed previously and should be cited, for example:

https://pubmed.ncbi.nlm.nih.gov/14657173/.

8. The dorsal hippocampus is generally regarded as more critical for spatial and episodic memory than the ventral hippocampus. Why were the experiments performed in ventral CA3? The authors themselves also note that dorsal and ventral CA3 may differ in their connectivity rules.

9.Fig. 2b: Were all transmission failures in the P45–50 group marked in the figure? How was transmission failure quantitatively defined? It would be better to quantify the transmission reliability. Fig. 2c: Statistical results comparing the differences in amplitude should be provided. Figs. 2b and 2c are not referenced in the main text.

10. In Fig. 3h, only the apical dendrites appear to lengthen, whereas the basal dendrites do not. Could this difference be because the stratum oriens (SO) layer does not thicken, while the stratum radiatum (SR) layer does? In Fig. 3j, the dendrites in the P45–50 group appear noticeably thicker. Does this difference reach statistical significance?

11. In Fig. 5e, the input resistance of pyramidal neurons significantly decreases across development. If this phenomenon has been reported previously in the hippocampus or cortex, those studies should be cited in the main text.

12. In the manuscript, "Changes in connectivity of the CA3 network may contribute to the developmental refinement of the spatial map, which involves an increase in the proportion of CA1 place cells and a rise in spatial coherence during maturation (Wills et al., 2010; Langston et al., 2010)". This reference should also be added here: https://pubmed.ncbi.nlm.nih.gov/39572591/.

13. In the manuscript, "This may contribute to the decline of average firing rate of hippocampal neurons (Supplementary Fig. 6; Wills et al., 2010; Langston et al., 2010)." An additional highly relevant reference for the decrease in firing rate during development is: https://pubmed.ncbi.nlm.nih.gov/39572591/.

14. The text states that "sparse and structured connectivity may represent the emergence of "engrams", i.e. the cellular, molecular, and structural changes underlying learning and memory (Josselyn and Tonegawa, 2020)." and that "In this framework, connectivity motifs will represent previously stored information ("engrams"), and therefore would be expected to emerge with experience over time". However, engram cells typically form only after the animal has undergone specific learning or experience, and under baseline conditions c-Fos expression is not normally observed. Were the animals exposed to a novel environment or fear-conditioning stimuli prior to slicing for in vitro electrophysiology? If not, it may be inappropriate to claim that the observed sparse and structured connectivity "represents the emergence of engrams." At most, one could state that such connectivity may support the formation of a more precise engram ensemble, rather than constituting the engram itself.

Reviewer #3

(Remarks to the Author)
The paper by Vargas-Barroso and co-workers is aimed at investigating the development of recurrent synaptic connections in the mouse area CA3. The authors report a reduction in the connection probability and axonal length over development but an increase in postsynaptic spine density. This paper therefore suggests an important pruning occurring in the area CA3 during development.
The paper is well written and the quality of the science is excellent and impressive. I have only a few points that require specific attention.

Specific points
1. Most of the changes appears between P7/8 and P18/25 while from P18/25 to P45/50 the modifications in the rate of connections, axon length…. are not significant. Does this mean that mouse CA3 region is adult at P18/25? What specifically occur at in the P8-P18 time period? Eyes opening that occurs at ~P13? This point should be further discussed.
2. How do the authors explain the discrepancy between the reduction in axonal length by 50% and the increase in both dendritic length (factor 4) and spine density (factor 4) from P7/8 to P45/50? Does it mean that at early stages CA3-CA3 synapses can be made directly on dendritic shaft/soma? If yes, electron microscopy would be helpful.

Minor points
-Introduction: Hebbian plasticity at CA3-CA3 synapses has been reported before 2016. Please, cite the pioneering papers.
-Supplementary Figure 2g, the unit (ms) is missing.
-Supplementary Figure 5 is very interesting and should be placed in the main set of figures.
-Supplementary Figure 6f. The units should be mV.ms-1 in both the graph and the traces.

Version 1:

Reviewer comments:

Reviewer #1

(Remarks to the Author)

Thank you to the authors for implementing many of my suggestions from the first review. The manuscript has improved in clarity. I have only one minor suggestion remaining.

• “Storage of patterns was accomplished via a clipped Hebbian synaptic plasticity rule.” Perhaps this should reference a Methods section or a Supp. Fig. that concisely explains this rule.

We thank the reviewer. We have added a reference to the Results (p. 8, center) and an explanation to the Methods section (p. 19, center of the revised version) as requested.

There should be a pointer to the Methods on P8.

(Remarks on code availability)

Reviewer #2

(Remarks to the Author)
I appreciate the authors’ careful efforts in addressing my previous questions and revising the manuscript accordingly. The revisions have substantially improved the clarity and rigor of the study. In particular, the modeling results are now more convincing and clearly presented. I have no further questions or concerns and believe the manuscript is now suitable for publication.

(Remarks on code availability)

Reviewer #3

(Remarks to the Author)
My initial remarks have been satisfactorily addressed in the revised version of this manuscript. I have no further comment.

(Remarks on code availability)

Open Access This Peer Review File is licensed under a Creative Commons Attribution 4.0 International License, which permits use, sharing, adaptation, distribution and reproduction in any medium or format, as long as you give appropriate credit to the original author(s) and the source, provide a link to the Creative Commons license, and indicate if changes were made.
In cases where reviewers are anonymous, credit should be given to 'Anonymous Referee' and the source.
The images or other third party material in this Peer Review File are included in the article’s Creative Commons license, unless indicated otherwise in a credit line to the material. If material is not included in the article’s Creative Commons license and your intended use is not permitted by statutory regulation or exceeds the permitted use, you will need to obtain

permission directly from the copyright holder.
To view a copy of this license, visit https://creativecommons.org/licenses/by/4.0/

Point-by-point reply to the reviewer's comments NCOMMS-25-65843
Reply to comments of Reviewer #1

In this paper, the authors performed simultaneous patch-clamp recordings from multiple CA3 pyramidal neurons in acute rodent brain slices at different developmental time points. They rigorously characterized morphological and functional features of the neurons and synaptic connections, and related changes in function to memory capacity with modeling. The scope of the study is very focused, the data is high-quality, and the conclusions are well-supported by the data.

We thank the reviewer for several positive comments about our paper ("rigorously", "focused", "high quality", "well supported"). Changes are highlighted in the text in yellow.

Below are suggestions to improve the manuscript.

P3: Here are additional relevant citations for connectivity structured by birth date:
- Druckmann S, Feng L, Lee B, Yook C, Zhao T, Magee JC, Kim J. Structured synaptic connectivity between hippocampal regions. Neuron. 2014 Feb 5;81(3):629-40.
- Huszár, R., Zhang, Y., Blockus, H. et al. Preconfigured dynamics in the hippocampus are guided by embryonic birthdate and rate of neurogenesis. Nat Neurosci 25, 1201–1212 (2022).

We thank the reviewer for the suggestions. We have added these citations as requested (p. 3, top of the revised manuscript).

P30, Fig 2a: Expressing the numbers of connections in these bar plots as found over tested would be more intuitive.

We thank the reviewer for the suggestion. We have made this modification as requested.

P30, Fig. 2b,c: These panels are not discussed in the text. The equation under Fig. 2b is not discussed in the figure legend.

We thank the reviewer for pointing this out. We have added a sentence to the text (p. 4, center of the revised version) and a statement to the figure legend (p. 32).

P34, Fig. 5a: Extra space between "Unitary" and "EPSP" in panel subtitle.

We thank the reviewer for picking this up. We have corrected this inconsistency.

P34, Fig. 5d: Inset is not described in legend.

We thank the reviewer for pointing this out. We have added a sentence to the legend (p. 37).

P35, Fig. 6e: I find the clipped spikes and the upward / downward arrows are not helping with understanding the figure. Perhaps just use two shaded bars of color to indicate where presynaptic stimulation vs postsynaptic current injection occurred, and show the full spike height in both cases.

We thank the reviewer for these valuable suggestions. We now show the full spikes, and also revised labeling of the figure to enhance clarity.

P8: • "synapses in the P7–8 age group were significantly more depressing." Perhaps this should reference Supp. Fig. 7e (P47) where you quantify short term adaptation of EPSP amplitude.

We thank the reviewer. We have added a reference to the figure as requested (p. 7, center of the revised version).

• "Storage of patterns was accomplished via a clipped Hebbian synaptic plasticity rule." Perhaps this should reference a Methods section or a Supp. Fig. that concisely explains this rule.

We thank the reviewer. We have added a reference to the Results (p. 8, center) and an explanation to the Methods section (p. 19, center of the revised version) as requested.

P9: • "decreased memory capacity, as the total number of synapses became reduced." But didn't the data show increased dendrite length and spine density? Is it possible in this model to separately control connection probability and number of synapses?

We thank the reviewer for this excellent comment. We attempted to follow the suggestion of the reviewer and tried to independently vary connectivity and synapse number. We have added new simulations in which we kept the number of synapses per cell constant (Supplementary Figure 10b of the revised paper). We need to point out that these changes may not be biologically meaningful, because the number of cells changes in such a simulation paradigm. We have added a caveat sentence to help the reader understand this point (p. 8, center of the revised version). We thank the reviewer for the useful suggestion.

• Given that different developmentally-regulated network parameters either increase or decrease storage capacity, is there a final conclusion about whether we expect the net difference to be an increase or a decrease by adulthood?

We agree with the reviewer that the changes are complex. Sparse connectivity can increase memory capacity when the number of synapses per cell is kept constant, nonrandom connectivity motifs may enhance the robustness of retrieval in the parameter space, and broadening of connectivity and elevation of firing threshold increase the absolute values of memory capacity. Thus, developmental changes improve different aspects of memory storage and retrieval. We have added a sentence to the discussion to clarify this point (p. 11, bottom of the revised manuscript).

P21: The provided explanation of the method to quantify pattern separation by a difference between input-input correlations and output-output correlations is insufficient. Please expand for clarity. It would be even better to provide a supplementary figure showing the scatter plots and illustrating the method. Also, a Guzman et al., 2021 is cited in line, but does not appear in the References. It is likely a typo referring to Guzman et al., 2016. That study does not completely explain the method either, and I couldn't find it along the citation chain from there either.

We thank the reviewer for detecting this omission. We have (1) included a schematic illustration of the analysis procedures in the new Supplementary Figure 9, (2) added input-output correlation plots and 3D plots of pattern similarity index (ψ) to Supplementary Figure 10, (3) added Guzman et al., 2021, Nature Computational Science to the reference list, and (4) revised the Methods section (p. 20, bottom of the revised manuscript).

Reply to comments of Reviewer #2

The recurrent connections in the hippocampal CA3 region are considered to be a key neural mechanism underlying pattern completion and memory retrieval. This study investigates how these recurrent connections in CA3 develop across different stages. The work provides important insights into infant learning and memory, as well as the function of CA3 in adulthood. The authors show that at P7–8, CA3 recurrent connectivity is relatively dense and synaptic weights are relatively strong. With development, at P18–25 and P45–50, both connection probability and synaptic strength decline. Computational modeling further demonstrates that these sparse recurrent connections can effectively support memory storage, retrieval, and pattern completion. Their findings also suggest that the emergence of the adult-like connectivity pattern in CA3, i.e., the disynaptic connectivity motif, is shaped by experience-dependent processes, rather than being entirely preconfigured. Overall, this study represents an important advance in our understanding of the development of the hippocampus and memory.

We thank the reviewer for the positive comments on our paper ("important insights", "important advance"). Changes are highlighted in the text in green.

I have some comments that should be addressed before publication:

1. About the choice of developmental stage groups: while P7–8 and P45–50 are appropriate for representing early postnatal and near-adult stages, respectively, the P18–25 interval spans a relatively long developmental period that encompasses several important milestones: grid cells emerge around P20–21, infantile amnesia exists at P17–18 and disappears at P24, and memory precision appears by ~P24 (https://pubmed.ncbi.nlm.nih.gov/40215964/). If feasible, it would be informative to subdivide this window into P18–20 and P23–25 (or use another reasonable subdivision) and assess at least one key metric (e.g., connection probability, synaptic strength, or spine density). Such a subdivision could help determine whether the observed developmental trend is genuinely gradual or instead reflects a more discrete transition. If the authors' intention is simply to demonstrate a monotonic, gradual refinement across P7–8, P18–25, and P45–50, rather than to emphasize a specific developmental period within P18–25, the use of the broader window is acceptable, but it should be explicitly acknowledged.

We thank the reviewer for the suggestion. We have subdivided the middle interval (P18–25) into two subintervals (P18–21, P22–25) as requested. We have also generated scatter plots of different parameters against developmental time and analyzed the data by correlation analysis. Data for EPSP potency and input resistance seem to show a stepwise change at around P20, but statistical significance is unclear. In any case, we have included the scatter plots as new Supplementary Figure 4. Finally, we have included citations to the paper by Ramsaran et al., 2025 as requested (p. 3 and 9 of the revised paper).

2. Relatedly, the text states: "episodic memory develops relatively late, approximately two years after birth. As this time point in humans is thought to correspond to the developmental range P14–21 in mice (Bevandić et al., 2024), these changes may be related to the developmental phenomena investigated in the present paper". This description is not entirely accurate. In rodents, the emergence of precise episodic-like memory formation is generally reported around P24, rather than P14–21 (https://pubmed.ncbi.nlm.nih.gov/40215964/).

The reviewer is totally correct. We have revisited the Bevandic et al., 2024 paper, which mentions " … a 3-week-old rodent may be comparable to a 2-year-old child in terms of memory maturation". We have revised our statements in the text, and also cited the paper by Ramsaran et al., 2025 (p. 12, top of the revised manuscript).

3. Regarding the model, the parameter "pattern similarity index" might require clarification with a schematic illustration: what are its precise inputs and outputs? In the text, the network input is described as "decorrelated information from the dentate gyrus". Although the mossy-fiber tract length appears unchanged across development, as shown in this study, its density may vary as the number of granule cells increases; consequently, the inputs to CA3 could differ across developmental stages.

We thank the reviewer for these suggestions. We have (1) included a schematic illustration of the analysis procedures in the new Supplementary Figure 9, (2) added input-output correlation plots and 3D plots of pattern similarity index (ψ) to Supplementary Figure 10, (3) added Guzman et al., 2021, Nature Computational Science to the reference list, and (4) revised the Methods section (p. 20, bottom of the revised manuscript).

4. In addition, regarding the model, the experimental results in this study have shown a trend for recurrent connectivity to be higher in CA3a/b than in CA3c, supporting the view that CA3a/b favors pattern completion while CA3c supports pattern separation (see https://pubmed.ncbi.nlm.nih.gov/26298277/ and https://pubmed.ncbi.nlm.nih.gov/26298276/). The present study reported a decrease in connection probability across these subregions with development. Why does the model predict that both pattern completion and pattern separation increase with development across CA3 subregions?

We thank the reviewer for this comment. We have performed additional simulations with moderately correlated patterns, and found that sparsification of connectivity slightly shifted the network in the direction from pattern completion to pattern separation

(Supplementary Figure 10e). Although this would be consistent with the predictions of the reviewer, we would like to point out that a simple relation between recurrent connectivity and pattern completion–pattern separation balance is not expected. First, the impact of the mossy fiber input may be important. If there was more input to CA3c, there could be more pattern separation (as separated input signals are "inherited" from the dentate gyrus). Second, inhibition and connectivity of inhibitory interneurons will be relevant, as lateral inhibition is a key mechanism for pattern separation (see Guzman et al., 2021, Nat. Comp. Sci.). Third, spatial gradients are not implemented in the model; this could affect the pattern completion–pattern separation balance. Fourth, CA3 cell diversity has to be taken into account. For example, CA3 is composed of superficial and deep cells, and only the former receive mossy fiber input (see Watson et al., 2025, Cell Reports). We have added an explanation, and also cited the two papers mentioned by the reviewer (Lu et al., 2015, Neuron; Lee et al., 2015, Neuron; p. 11, bottom of the revised manuscript). Finally, the trend for connectivity to be higher in CA3 a / b was only a nonsignificant trend, as perfectly summarized by the reviewer. Thus, we cannot derive any major conclusions from this observation.

5. The authors propose that the observed connectivity pattern in CA3 is sculpted by experience rather than predetermined, noting that early in development, connectivity is relatively dense, whereas disynaptic motifs are rare, compared to later developmental stages. However, an alternative possibility is that the connectivity observed at P7–8 is largely predetermined, and that the motifs seen later simply reflect those that remain after developmental elimination—arising from a combination of both innate programs and experience, rather than experience alone. If experience were the sole contributor, one would expect these motifs to be formed de novo rather than selectively eliminated from an initially dense network. The authors might therefore consider discussing how to distinguish between selective elimination and de novo formation (for example, by longitudinal imaging).

We thank the reviewer for these excellent suggestions. We agree that the two possibilities represent extreme scenarios, and that combinations are always possible. To address this possibility, we would have to test whether neurons connected by motifs in the mature network were previously connected in the young network or not. This would require a longitudinal analysis at the single-synapse level, which is currently not feasible. In any case, we have included this possibility in the discussion, as requested by the reviewer (p. 10, bottom of the revised manuscript).

6. This study demonstrated a developmental decrease in connection probability, a pruning of short-distance recurrent synapses, and a decrease in transmission efficacy. Some mechanistic speculation should be provided. One plausible contributor is the maturation of inhibition. Parvalbumin-positive (PV) interneurons in CA3, as in other hippocampal subfields, undergo significant maturation during development (https://pubmed.ncbi.nlm.nih.gov/28154241/).

We thank the reviewer for this comment. We mention different possible mechanisms, including a potential role of PV^+ interneurons as requested (p. 10, center of the revised manuscript). We also cite earlier papers (Donato et al., 2013; Miranda et al., 2023).

7. The authors report a developmental decline of “detonating” (high-fidelity, single-spike-driven) synaptic transmission in CA3. A similar phenomenon has been observed previously and should be cited, for example: https://pubmed.ncbi.nlm.nih.gov/14657173/.

We thank the reviewer for drawing our attention to the Meredith et al., 2003 paper, which nicely examines plasticity rules at different developmental time points. However, there are multiple differences between this paper and the present study. Our recordings report unitary EPSPs generated by activation of a single presynaptic cell in the absence of any plasticity induction protocol, whereas the Meredith et al., 2003 paper reports stimulation of multiple inputs by fiber tract stimulation and examines different plasticity induction paradigms. Some of the EPSPs after LTP induction are large, so we might expect that they trigger spikes under certain conditions. However, there is no demonstration of such a “detonation” in this paper. Miles and Wong, 1986, provided anecdotic evidence for detonating CA3–CA3 synapses in young guinea-pigs. We agree that this should be acknowledged in our paper, which we have now done (p. 7, bottom of the revised version).

8. The dorsal hippocampus is generally regarded as more critical for spatial and episodic memory than the ventral hippocampus. Why were the experiments performed in ventral CA3? The authors themselves also note that dorsal and ventral CA3 may differ in their connectivity rules.

We thank the reviewer for this comment. Our slices were cut from the center of the hippocampus with a bias towards the ventral side – in that sense our previous statement on p. 4 was not entirely correct. To provide a better quantitative measure of slice location, we drew a line along the cashewnut-like structure of the hippocampus, and projected our quasi-transverse slice planes onto this ventral-to-dorsal axis. For these measurements, we used the Allen Brain Atlas DAPI staining in 8- to 12-week-old mice, comparing them with Nissl/DAPI stainings in our own slices. The majority of slices fell into a ventral-to-dorsal distance range of ~4000–4500 μm. For a total ventral-to-dorsal axis length of ~10,000 μm, this indicates a bias towards ventral hippocampus. We have now included these data in the Methods section (p. 13, top of the revised version).

9.Fig. 2b: Were all transmission failures in the P45–50 group marked in the figure? How was transmission failure quantitatively defined? It would be better to quantify the transmission reliability. Fig. 2c: Statistical results comparing the differences in amplitude should be provided. Figs. 2b and 2c are not referenced in the main text.

We thank the reviewer for these valuable comments. (1) Failures were defined „as a trace in which the peak amplitude of the response to the presynaptic AP was less than three times the standard deviation of the preceding baseline“ (p. 17, bottom). This is now repeated in the legend for clarity (p. 32 of the revised manuscript). (2) Statistics

have been added to the figure legend (p. 32 of the revised paper). (3) Fig. 2b and 2c are now referenced in the text (p. 4, center of the revised manuscript).

10. In Fig. 3h, only the apical dendrites appear to lengthen, whereas the basal dendrites do not. Could this difference be because the stratum oriens (SO) layer does not thicken, while the stratum radiatum (SR) layer does? In Fig. 3j, the dendrites in the P45–50 group appear noticeably thicker. Does this difference reach statistical significance?

We thank the reviewer for this comment. We have measured the thickness of stratum oriens and stratum radiatum and found that both layers show an increase in their absolute thickness, which is statistically significant among the age groups. We further analyzed the SR to SO size ratio and found that the latter is similar throughout development, with a ratio of 1 : 0.48 for P7–8; 1 : 0.5 for P18–25 and 1 : 0.54 for P45–50. Thus, the microscopic changes in dendrite length cannot be easily explained by macroscopic changes in layer thickness. We have also analyzed dendrite diameter, as requested. The results reveal a significant increase of dendritic diameter during postnatal development. The data have been included in Fig. 3, along with the description of analysis procedures in the Methods section (p. 16, center of the revised paper).

11. In Fig. 5e, the input resistance of pyramidal neurons significantly decreases across development. If this phenomenon has been reported previously in the hippocampus or cortex, those studies should be cited in the main text.

We thank the reviewer for raising this point. We have added a relevant citation (Tyzio et al., 2003) to our manuscript.

12. In the manuscript, “Changes in connectivity of the CA3 network may contribute to the developmental refinement of the spatial map, which involves an increase in the proportion of CA1 place cells and a rise in spatial coherence during maturation (Wills et al., 2010; Langston et al., 2010)”. This reference should also be added here: https://pubmed.ncbi.nlm.nih.gov/39572591/.

We thank the reviewer for the reminder. We have added a reference to the Wang et al., 2024 paper as requested.

13. In the manuscript, “This may contribute to the decline of average firing rate of hippocampal neurons (Supplementary Fig. 6; Wills et al., 2010; Langston et al., 2010).” An additional highly relevant reference for the decrease in firing rate during development is: https://pubmed.ncbi.nlm.nih.gov/39572591/.

We have cited the Wang et al., 2024 paper in the revised version as requested.

14. The text states that “sparse and structured connectivity may represent the emergence of “engrams”, i.e. the cellular, molecular, and structural changes underlying learning and memory (Josselyn and Tonegawa, 2020).” and

that "In this framework, connectivity motifs will represent previously stored information ("engrams"), and therefore would be expected to emerge with experience over time". However, engram cells typically form only after the animal has undergone specific learning or experience, and under baseline conditions c-Fos expression is not normally observed. Were the animals exposed to a novel environment or fear-conditioning stimuli prior to slicing for in vitro electrophysiology? If not, it may be inappropriate to claim that the observed sparse and structured connectivity "represents the emergence of engrams." At most, one could state that such connectivity may support the formation of a more precise engram ensemble, rather than constituting the engram itself.

We thank the reviewer for this comment. Animals were not exposed to novel environments or fear-conditioning stimuli. However, cages typically contained mother, littermates, and complex nesting material (cotton-like and cardboard). This is now mentioned in the manuscript (p. 12, bottom of the revised version). As animals grow up, such environments will appear novel. Regardless, we agree with the reviewer that it may be appropriate to attenuate our statements and replace "represents the emergence of engrams" by "sparse and structured connectivity might be part of the "engram" (p. 10, bottom of the revised version).

Reply to comments of Reviewer #3

The paper by Vargas-Barroso and co-workers is aimed at investigating the development of recurrent synaptic connections in the mouse area CA3. The authors report a reduction in the connection probability and axonal length over development but an increase in postsynaptic spine density. This paper therefore suggests an important pruning occurring in the area CA3 during development. The paper is well written and the quality of the science is excellent and impressive. I have only a few points that require specific attention.

We thank the reviewer for several positive comments on our paper ("well written", "excellent", "impressive", "only a few points"). Changes are highlighted in the text in cyan.

Specific points

1. Most of the changes appears between P7/8 and P18/25 while from P18/25 to P45/50 the modifications in the rate of connections, axon length…. are not significant. Does this mean that mouse CA3 region is adult at P18/25? What specifically occur at in the P8-P18 time period? Eyes opening that occurs at ~P13? This point should be further discussed.

We thank the reviewer for this suggestion. The changes may very well be related to onset of hearing (P12) and eye opening (Benoit et al., 2015), as well as the sensitive period for episodic memory formation (Ramsaran et al., 2025), as implied by the reviewer's comment. We now briefly discuss this in the manuscript as requested (p. 9, center of the revised version).

2. How do the authors explain the discrepancy between the reduction in axonal length by 50% and the increase in both dendritic length (factor 4) and spine density (factor 4) from P7/8 to P45/50? Does it mean that at early stages CA3-CA3 synapses can be made directly on dendritic shaft/soma? If yes, electron microscopy would be helpful.

We thank the reviewer for this excellent comment. A change in the proportion of spine versus shaft synapses is certainly a possibility. Boyer et al., 1998, found that the proportion of shaft synapses decreases from 64% to 18% during development. A second possibility is that a change between local and long-range connections takes place. Proximal collaterals will prevail in the young, whereas distal collaterals will contribute more in older animals. We now better discuss these possibilities in our paper, including a reference to Boyer et al., 1998 (p. 10, top of the revised manuscript).

Minor points

-Introduction: Hebbian plasticity at CA3-CA3 synapses has been reported before 2016. Please, cite the pioneering papers.

We thank the reviewer for this comment. We have added a citation to Debanne et al., 1998 in slice culture as an earlier demonstration.

-Supplementary Figure 2g, the unit (ms) is missing.

We thank the reviewer for pointing this out. We have corrected the mistake.

-Supplementary Figure 5 is very interesting and should be placed in the main set of figures.

We thank the reviewer for appreciating our results. After careful consideration, we decided not to include this figure in the main body of the manuscript. We feel that incorporating this figure would introduce redundancy to Fig. 4, which we would like to avoid. We hope the reviewer finds this acceptable.

-Supplementary Figure 6f. The units should be mV.ms-1 in both the graph and the traces.

We thank the reviewer for pointing this out. We have corrected the mistake.

Once again, we would like to thank the reviewers for helping us to further improve our paper.

References

Allen Reference Atlas – Mouse Brain [brain atlas]. Available from atlas.brain-map.org.

Boyer C, Schikorski T, Stevens CF. Comparison of hippocampal dendritic spines in culture and in brain. J Neurosci. 1998 Jul 15;18(14):5294–5300. doi: 10.1523/JNEUROSCI.18-14-05294.1998. PMID: 9651212.

Debanne D, Gähwiler BH, Thompson SM. Long-term synaptic plasticity between pairs of individual CA3 pyramidal cells in rat hippocampal slice cultures. J Physiol. 1998 Feb 15;507 (Pt 1)(Pt 1):237–247. doi: 10.1111/j.1469-7793.1998.237bu.x. PMID: 9490845.

Donato F, Rompani SB, Caroni P. Parvalbumin-expressing basket-cell network plasticity induced by experience regulates adult learning. Nature. 2013 Dec 12;504(7479):272–276. doi: 10.1038/nature12866. PMID: 24336286.

Druckmann S, Feng L, Lee B, Yook C, Zhao T, Magee JC, Kim J. Structured synaptic connectivity between hippocampal regions. Neuron. 2014 Feb 5;81(3):629–640. doi: 10.1016/j.neuron.2013.11.026. PMID: 24412418.

Guzman SJ, Schlögl A, Espinoza C, Zhang X, Suter BA, Jonas P. How connectivity rules and synaptic properties shape the efficacy of pattern separation in the entorhinal cortex-dentate gyrus-CA3 network. Nat Comput Sci. 2021 Dec;1(12):830–842. doi: 10.1038/s43588-021-00157-1. PMID: 38217181.

Huszár R, Zhang Y, Blockus H, Buzsáki G. Preconfigured dynamics in the hippocampus are guided by embryonic birthdate and rate of neurogenesis. Nat Neurosci. 2022 Sep;25(9):1201–1212. doi: 10.1038/s41593-022-01138-x. PMID: 35995878.

Lee H, Wang C, Deshmukh SS, Knierim JJ. Neural population evidence of functional heterogeneity along the CA3 transverse axis: Pattern completion versus pattern separation. Neuron. 2015 Sep 2;87(5):1093–1105. doi: 10.1016/j.neuron.2015.07.012. Epub 2015 Aug 19. PMID: 26298276.

Lu L, Igarashi KM, Witter MP, Moser EI, Moser MB. Topography of place maps along the CA3-to-CA2 axis of the hippocampus. Neuron. 2015 Sep 2;87(5):1078–1092. doi: 10.1016/j.neuron.2015.07.007. PMID: 26298277.

Miranda JM, Cruz E, Bessières B, Alberini CM. Hippocampal parvalbumin interneurons play a critical role in memory development. Cell Rep. 2022 Nov 15;41(7):111643. doi: 10.1016/j.celrep.2022.111643. PMID: 36384113.

Ramsaran AI, Ventura S, Gallucci J, De Snoo ML, Josselyn SA, Frankland PW. A sensitive period for the development of episodic-like memory in mice. Curr Biol. 2025 May 5;35(9):2032–2048.e3. doi: 10.1016/j.cub.2025.03.032. Epub 2025 Apr 10. PMID: 40215964.

Tyzio R, Ivanov A, Bernard C, Holmes GL, Ben-Ari Y, Khazipov R. Membrane potential of CA3 hippocampal pyramidal cells during postnatal development. J Neurophysiol. 2003 Nov;90(5):2964–2972. doi: 10.1152/jn.00172.2003. PMID: 12867526.

Wang C, Yang H, Chen S, Wang C, Chen X. Early and late place cells during postnatal development of the hippocampus. Nat Commun. 2024 Nov 21;15(1):10075. doi: 10.1038/s41467-024-54320-z. PMID: 39572591.

Point-by-point reply to the reviewer's comments on NCOMMS-25-65843A

Reviewer #1

"Thank you to the authors for implementing many of my suggestions from the first review. The manuscript has improved in clarity. I have only one minor suggestion remaining. • "Storage of patterns was accomplished via a clipped Hebbian synaptic plasticity rule." Perhaps this should reference a Methods section or a Supp. Fig. that concisely explains this rule. We thank the reviewer. We have added a reference to the Results (p. 8, center) and an explanation to the Methods section (p. 19, center of the revised version) as requested. There should be a pointer to the Methods on P8."

We thank the reviewer for appreciating our efforts. We have added a reference to the Methods section on p. 8 as requested.

Reviewer #2

"I appreciate the authors' careful efforts in addressing my previous questions and revising the manuscript accordingly. The revisions have substantially improved the clarity and rigor of the study. In particular, the modeling results are now more convincing and clearly presented. I have no further questions or concerns and believe the manuscript is now suitable for publication."

We thank the reviewer for the positive comments ("careful", "clarity and rigor", "convincing") and for supporting publication of our paper.

Reviewer #3

"My initial remarks have been satisfactorily addressed in the revised version of this manuscript. I have no further comment."

We thank the reviewer for the positive comments ("satisfactorily") and for supporting publication of our paper.
